# Multifunctional Double-negative T Cells in Sooty Mangabeys Mediate T-helper Functions Irrespective of SIV Infection

**DOI:** 10.1371/journal.ppat.1003441

**Published:** 2013-06-27

**Authors:** Vasudha Sundaravaradan, Ramsey Saleem, Luca Micci, Melanie A. Gasper, Alexandra M. Ortiz, James Else, Guido Silvestri, Mirko Paiardini, John D. Aitchison, Donald L. Sodora

**Affiliations:** 1 Seattle Biomedical Research Institute, Seattle, Washington, United States of America; 2 Institute for Systems Biology, Seattle, Washington, United States of America; 3 Yerkes National Primate Research Center, Emory University, Atlanta, Georgia, United States of America; National Institute of Allergy and Infectious Diseases, National Institutes of Health, United States of America

## Abstract

Studying SIV infection of natural host monkey species, such as sooty mangabeys, has provided insights into the immune changes associated with these nonprogressive infections. Mangabeys maintain immune health despite high viremia or the dramatic CD4 T cell depletion that can occur following multitropic SIV infection. Here we evaluate double-negative (DN)(CD3+CD4−CD8−) T cells that are resistant to SIV infection due to a lack of CD4 surface expression, for their potential to fulfill a role as helper T cells. We first determined that DN T cells are polyclonal and predominantly exhibit an effector memory phenotype (CD95+CD62L−). Microarray analysis of TCR (anti-CD3/CD28) stimulated DN T cells indicated that these cells are multifunctional and upregulate genes with marked similarity to CD4 T cells, such as immune genes associated with Th1 (IFNγ), Th2 (IL4, IL5, IL13, CD40L), Th17 (IL17, IL22) and T_FH_ (IL21, ICOS, IL6) function, chemokines such as CXCL9 and CXCL10 and transcription factors known to be actively regulated in CD4 T cells. Multifunctional T-helper cell responses were maintained in DN T cells from uninfected and SIV infected mangabeys and persisted in mangabeys exhibiting SIV mediated CD4 loss. Interestingly, TCR stimulation of DN T cells from SIV infected mangabeys results in a decreased upregulation of IFNγ and increased IL5 and IL13 expression compared to uninfected mangabeys. Evaluation of proliferative capacity of DN T cells *in vivo* (BrDU labeling) indicated that these cells maintain their ability to proliferate despite SIV infection, and express the homeostatic cytokine receptors CD25 (IL2 receptor) and CD127 (IL7 receptor). This study identifies the potential for a CD4-negative T cell subset that is refractory to SIV infection to perform T-helper functions in mangabeys and suggests that immune therapeutics designed to increase DN T cell function during HIV infection may have beneficial effects for the host immune system.

## Author Summary

SIV infection of sooty mangabeys is generally characterized by maintained CD4 T cell levels and a lack of disease progression despite active virus replication. We have previously shown however, that dramatic loss of CD4 T cells can occur during SIV infection of mangabeys. This study investigates the potential for double negative (DN) T cells (which lack CD4 and CD8, and are refractory to SIV/HIV infection) to perform helper T cell functions. In our study, sooty mangabey DN T cells exhibited a memory phenotype and a diverse repertoire in their T cell receptors. Once stimulated, the DN T cells expressed multiple cytokines, indicating that they have the potential to function as helper T cells (a function normally undertaken by CD4+ T cells). In SIV infected mangabeys, DN T cells continue to function, proliferate *in vivo*, and maintain expression of homeostatic cytokine receptors on their surface. It is therefore likely that DN T cells have the potential to compensate for the loss of CD4 T cells during SIV infection. These studies indicate that increasing DN T cell levels and/or function during pathogenic HIV infection may provide one tangible component of a functional cure, and inhibit progression to clinical disease and AIDS

## Introduction

While simian immunodeficiency virus (SIV) infection of Asian macaques generally results in progression to simian AIDS, SIV infection of African monkey species is typically associated with a nonpathogenic outcome. These African monkeys, including sooty mangabeys, are found naturally infected with SIV and are thought to have evolved with their species-specific viruses [1]. Studies of SIV infections of mangabeys and African green monkeys have provided key insights into the evolutionary mechanisms enabling these monkey species to remain free of disease. A number of studies have established that plasma viral levels are similar between natural SIV hosts and pathogenic infections observed in Rhesus macaques and HIV infected patients [2]–[6]. The SIV-specific antibody and cytotoxic T-lymphocyte (CTL) levels are also generally similar between the hosts of natural and pathogenic infections, indicating that stronger or more effective adaptive immune responses are not responsible for the non-pathogenic disease course [7], [8]. A major difference between natural and pathogenic infection is the lack of systemic immune activation (measured by proliferation/activation of immune cells, plasma cytokine levels) in the natural hosts during the chronic phase of the infection [4], [9]–[11]. How the natural hosts are able to suppress activation following the acute infection phase and why the natural hosts do not progress to simian AIDS despite high levels of viral replication are current avenues of research to understand SIV/HIV infection and host response.

While it is clear that helper T cells play a key role in the immune responses elicited in sooty mangabeys and other natural host infections [12], [13], there is evidence that preservation of specific helper T cell subsets may be associated with improved disease outcomes [14]. Recent studies have indicated that low levels of direct virus infection in central memory CD4+ T cells, correlate with the lack of disease progression in SIV infected mangabeys [13]. Although CD4 T cells are critical for immune health, healthy SIV+ mangabeys with extremely low CD4 T cell levels are found in the Yerkes mangabey colony [11]. In addition, studies from our laboratory have identified a dramatic CD4 T cell loss (<50 cells/µl of blood) within a cohort of experimentally SIV infected mangabeys, which have remained free of clinical AIDS for greater than 11 years despite this paucity of CD4+ T cells [15], [16]. Here, these SIV+ mangabeys are termed “CD4-low” to indicate their unique CD4 status. Throughout the text, the terms “CD4-healthy (healthy range for CD4 T cells, 400–1500 cells/µl of blood)” and “CD4-low” are used to indicate the CD4 status, although both groups remain asymptomatic for simian AIDS. It is likely that, similar to HIV infection, the expansion in co-receptor usage in the replicating virus of the SIV+ CD4-low mangabeys enables the infection and depletion of a greater number of CD4+ T cells [15], [16]. Despite this depletion, the CD4-low SIV infected mangabeys maintain low levels of systemic immune activation, preserve lymphoid architecture, preserve function of non CD4 T cells and most importantly, show no clinical signs of simian AIDS [15], [16]. We have also previously shown that these SIV infected CD4-low animals have robust SIV specific T cell responses, SIV specific antibodies and antigen specific responses to neo-antigens present in vaccines [15], [16]. We postulate that helper T cell function in SIV infected mangabeys is compensated for by a SIV-resistant T cell subset that lacks CD4 expression, the double negative T cell.

Double negative (DN) T cells are defined by expression of the CD3 protein, and a lack of both CD4 and CD8. Although the precise path of peripheral DN T cell development is not known, there are three models that have been proposed to explain how these cells arise and are maintained in the periphery [17]. One model proposes that immature DN thymocytes acquire expression of the T-cell receptor (TCR), bypass the subsequent double-positive (DP) and single-positive (SP) stages of classical T cell maturation, and migrate directly to the periphery. A second model suggests that a pre-T cell experiences all the normal development in the thymus but due to strong TCR∶MHC binding (sufficient to evade apoptosis) during negative selection, does not experience the CD4 or CD8 single positive stage. A third model suggests T cells proceeding all the way to the single-positive CD4 T cell, then experiencing a subsequent down-modulation of expression of the CD4 mRNA transcription and surface protein expression (reviewed in [17]).

Peripheral DN T cells have important functions in a wide range of different disease states in both mice and humans [15], [18]–[28]. In infectious disease mouse models, DN T cells produce IL17 early during pulmonary *Francisella tularensis* live vaccine strain infection and also secrete IFNγ necessary for controlling intracellular bacterial growth [20]. In humans, DN T cells play T helper roles during parasitic infection, expressing IFNγ, TNFα and IL17 as a component of the immune response to *Trypanosoma cruzi* [25]. In addition to T helper functional roles, the regulatory potential of DN T cells has been identified through their ability to inhibit antigen specific CD4 and CD8 T cell proliferation *in vitro* in healthy humans [28], [29]. Furthermore, higher DN T cells numbers early in HIV infection is associated with decreased chronic immune activation later in infection, suggesting a regulatory role for DN T cells in HIV+ patients [30]. The cytotoxic potential of DN T cells has also been demonstrated by their ability kill allogeneic as well as antigen-loaded syngeneic DCs [31], autoreactive CD8+ T cells [32], and activated allogeneic and syngeneic B cells [21]. Together, these data suggest that DN T cells in mice and humans exhibit functions similar to other T cell subsets.

In natural host monkey species, two different T cell subsets that lack a CD4 molecule have been described: the first is CD3+CD4−CD8α^dim^ cells [33], [34] and the second is CD3+CD4−CD8− DN T cells [15], [34] (these are distinct from invariant chain NKT cells [35]). In addition to peripheral blood, DN T cells are also present in different immunological tissue sites including lymph nodes, lungs, and rectal mucosa [15], [16], [36], [37]. These tissue sites also maintain DN T cell numbers as CD4+ T cells are depleted during both pathogenic and non-pathogenic SIV infections [15], [16], [36], [37].

Vinton et al., performed a cross sectional analysis of DN T cells in different natural hosts to elucidate their function and revealed that DN T cells are found in larger proportions (10–40% of lymphocytes) in natural hosts (sooty mangabeys, African green monkeys and patas monkeys) than in pathogenic host species (Rhesus macaques) [34]. In addition, there is limited apoptosis in DN T cells during SIV infection of natural hosts (sooty mangabeys) compared to SIV infected Rhesus macaques [38]. In these studies, DN T cells in the peripheral blood were predominantly memory cells with the majority of these cells having a central memory phenotype (expressing CD28, CD95 and CCR7). Assessment of purified populations also found very low, or undetectable, levels of SIV nucleic acid within the DN T cell subset, indicating that these cells are refractory to SIV infection [34]. These key pieces of data led us to hypothesize that DN T cells in natural hosts may be important for providing helper T cell responses during SIV infection, as they are more abundant in natural hosts, and do not become depleted during lentiviral infections.

The findings presented herein provide an assessment of the functional attributes of DN T cells in uninfected and SIV infected sooty mangabeys. Our previous studies have indicated that DN T cells may have specific T helper roles in the CD4-low mangabeys [15]. Using comprehensive transcriptome analysis of DN, CD4 and CD8 T cells, we now demonstrate that DN T cells in sooty mangabeys share a functional profile closer to CD4 T cells than to CD8s. In addition, by comparing SIV infected mangabeys with different CD4 T cells levels (CD4-low and CD4-healthy), we demonstrate that neither SIV infection nor SIV induced CD4-T cell loss markedly altered overall DN T cell function in this natural host model. We further demonstrate that, like CD4 T cells [13], DN T cells have a diverse T cell receptor repertoire and are predominantly effector memory cells that can be restricted by either MHC-I or MHC-II molecules (with a multi-functional response being associated with MHC-II utilization). The similarities between DN and CD4 T cells during SIV infection of sooty mangabeys indicate a key importance of this T cell subset and underscore the potential for this cell population as an immunotherapeutic target to prevent HIV-induced disease progression.

## Results

### Memory subset analysis demonstrated a predominantly effector memory phenotype for double negative T cells

**Figure 1:**
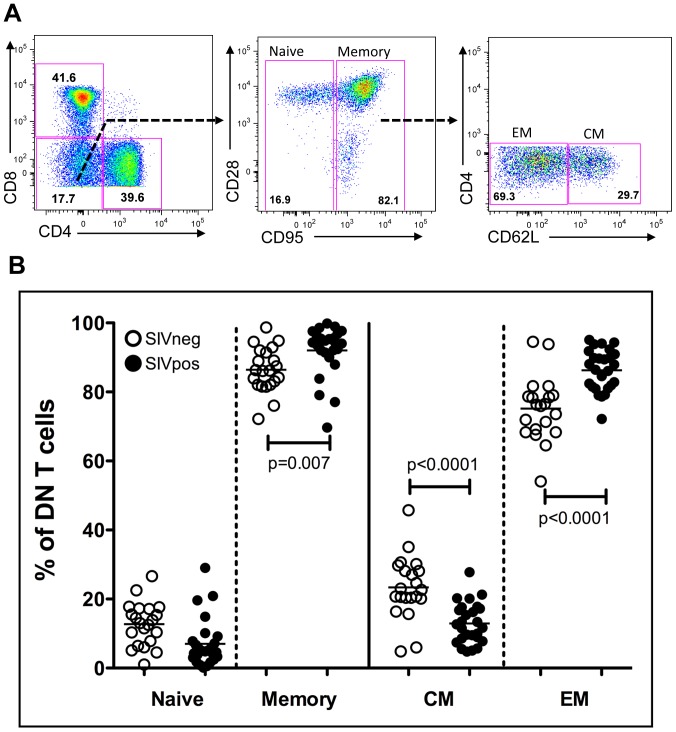
Double negative (CD3+CD4−CD8−) T cells from sooty mangabeys are predominantly effector memory cells. (A) Phenotypic assessment of T cell subsets (CD3+) depicting DN T cells (CD4−CD8− in the lower left quadrant) in sooty mangabey PBMC. Further assessment of naïve (CD95−) and memory DN subsets were performed based on CD95 and CD28 expression. Characterization of central memory (CM: CD95+CD62L+) and effector memory (EM: CD95+CD62L−) DN T cells are depicted. (B) DN T cells from uninfected mangabeys (open symbols) and SIV-infected mangabeys (filled symbols) were evaluated as Naïve, memory, CM and EM subsets and the relative percentage of each is depicted. DN T cells in the PBMCs are primarily memory cells (CD95+) with a predominant effector memory phenotype.

Double negative (DN) T cells express CD3 protein but do not express either CD4 or CD8 proteins and can be identified through flow cytometric analysis of peripheral blood cells (Figure 1A) as well as other tissues [15], [16] (Figure S2). This DN phenotype (absence of CD4 and CD8) can be observed at both the protein (Figure S1A) and mRNA levels (Figure S1C, depicts an absence of CD4 mRNA) and is maintained even after the cells are stimulated through their T-cell receptor (TCR). These cells are predominately not NKT cells, as staining with an canonical NKT cell-specific TCR antibody (anti-Vα24) indicated that less than 1% of DN T cells express this TCR (Figure S1A). In addition, mangabey DN T cells predominately express an αβTCR, with 17% expressing the γδTCR (similar between SIV infected and uninfected mangabeys (Figure S1B). In addition to peripheral blood, we also assessed the levels of DN T cells in mucosal tissues. The relative proportion of DN T cells (as a percentage of CD3+ cells) increased within the rectal biopsy samples during the times when the CD4 T cell levels decline within the SIV-infected CD4-low mangabeys (Figure S2A). In contrast, the proportion of DN T cells within the bronchoalveolar lavage (BAL) samples remain constant during this time, possibly due to an increased percentage of CD8 T cells in this site (Figure S2B). The naïve and memory phenotypes of the DN T cells can be categorized based on the expression of CD95. Although previous studies have further characterized central and effector memory subsets using the differential expression of the co-stimulatory marker CD28 [15], we have now adapted a more recent definition of identifying central memory (CD95+/CD62L+) and effector memory (CD95+/CD62L−) cell subsets [13]. CD62L expression on a T cell induces lymph node homing for the central memory T cells, whereas effector memory cells lose the CD62L expression upon recognition by cognate antigen in order to travel to the site of injury [13], [39], [40]. In the peripheral blood, DN T cells were predominantly memory cells (CD95+) with 75%±9% (mean±SD) of memory cells demonstrating an effector memory (EM: CD95+CD62L−) phenotype, and 25%±9% (mean±SD) with a central memory (CM: CD95+ CD62L+) phenotype. During chronic SIV infection, we observed an increase in the relative percentage of EM DN T cell population, possibly due to a conversion of CM to EM phenotype. Together, these findings identify a predominately memory phenotype for mangabey peripheral blood DN T cells, similar to human DN T cells (defined by the expression of CD45RO) [41].

### T cell repertoire in DN T cells

The T cell repertoire represents the potential for a T cell population to recognize a broad range of antigens, and provide protection against more potentially pathogenic organisms. To evaluate the diversity of purified mangabey DN T cells we utilized PCR primer sets based on Rhesus macaque TCR designed to potentially amplify 23 Vβ sequences. Multiple Vβ sequences were amplified from DN T cells from uninfected, SIV infected CD4-healthy and SIV infected CD4-low mangabeys (Table 1). A further evaluation of the length heterogeneities of the CDR3 region was undertaken by spectratyping [42], [43] to determine the intra-Vβ diversity (Figure S3). Five Vβ genotypes were not detected in DN and CD4 T cells irrespective of SIV infection status. This lack of detection occurred even though these five Vβ sequences share 100% homology in the Rhesus and human genomes (therefore indicating a high likelihood of nucleotide similarity in mangabeys as well) and may thus indicate a the absence of these Vβs in the mangabey repertoire (although the potential for primer mismatch to the mangabey sequences can not be ruled out). In the detected Vβ genotypes, polyclonal repertoire was observed in the DN T cells with 11 Vβ amplified in DN T cells from uninfected, 17 Vβ in SIV infected CD4-healthy and 14 Vβ for the SIV infected CD4low mangabeys (Table 1). This repertoire was similar to the repertoire of CD4 T cells in the same animals (data not shown). Junctional diversity within the majority Vβ CDR3s was identified through a visualization of DNA length differences (3 bps intervals), with the most prevalent CDR3 length generally at the central position (Figure S3, Table S1). Junctional diversity was observed for each amplified Vβ with the exception of Vβ7 and Vβ20, which manifest as single peaks (indicating clonality) in the uninfected mangabeys (Table 1). Interestingly, 4 Vβ genotypes undetectable in the uninfected mangabeys (Vβ 8, 15, 21 and 24) were present in SIV infected mangabeys (both CD4-healthy and CD4 low. Table 1) providing evidence for expansion of T cells with TCR containing these Vβ regions after SIV infection. These findings indicate that DN T cells express a diverse polyclonal TCR repertoire in both uninfected and SIV infected mangabeys, during both CD4-healthy and CD4 T cell depleting conditions.

**Table 1:** Spectratyping analysis of DN T cells from SIV infected and uninfected mangabeys.

Vβ	Junctional diversity	Uninfected	SIV+ CD4-healthy	SIV+ CD4-low
2	5	+	+	+
3	7	+	+	+
5	5	+	+	+
6	7	+	+	+
7A	5	+^a^	+	+
8	9	−	+	+
9	6	+	+	+
11	6	+	−	−
12B	7	+	+	+
13	7	+	+	+
15	7	−	+	+
16	6	−	+	−
18	4	−	+	−
20	5	+^a^	+	+
21	6	−	+	+
22	7	+	+	+
23	7	−	+	−
24	7	−	+	+

+ indicates detected Vβ, — indicates undetected Vβ,

a indicates specific Vβ had single clonally expanded peak.

### Transcriptome analysis of CD4, CD8 and DN T cells from uninfected mangabeys identifies similarities between DN and CD4 T cells

**Figure 2:**
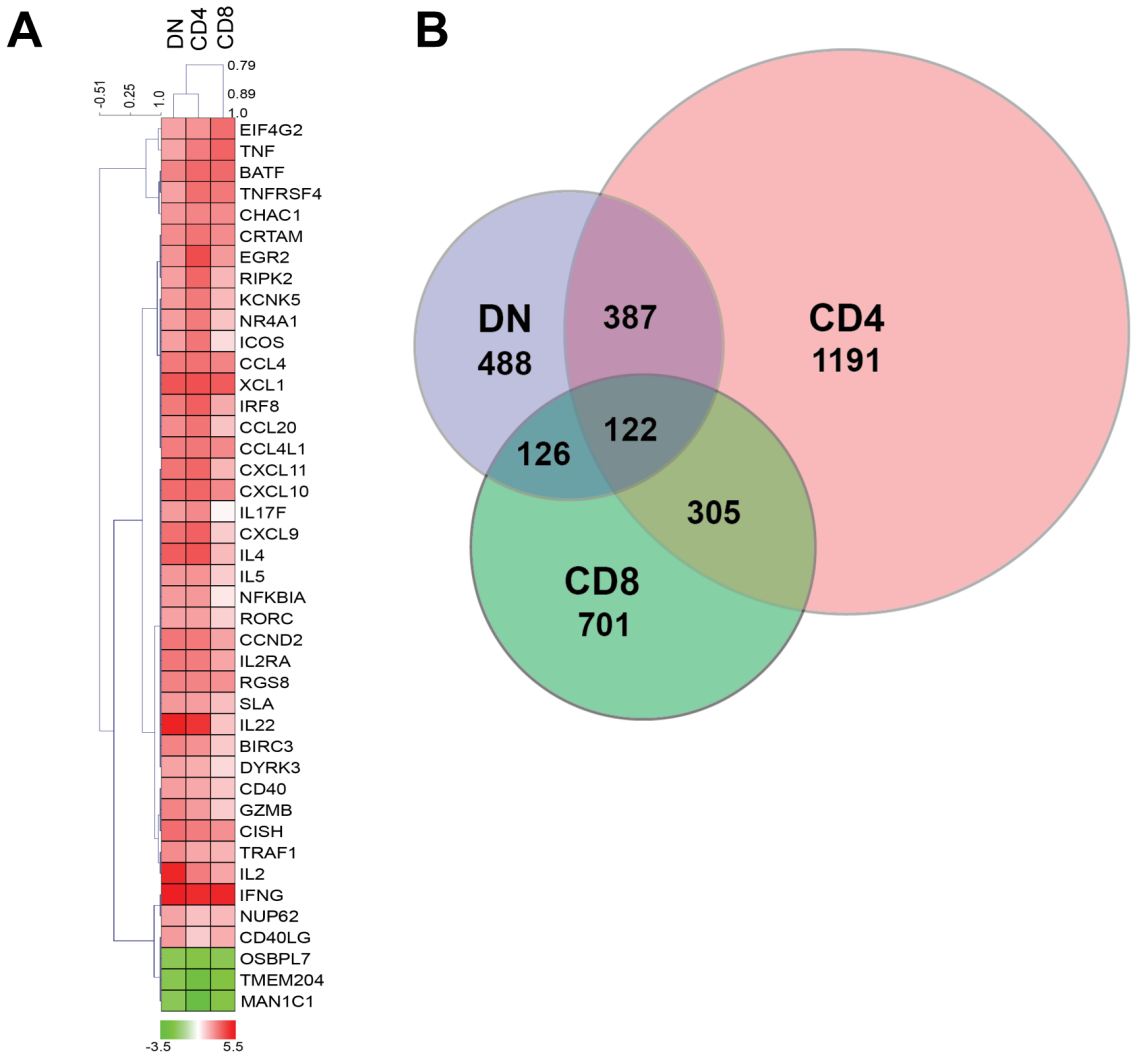
Transcriptome analysis utilizing microarray of stimulated CD4, CD8 and DN T cells from uninfected mangabeys demonstrates relatedness of CD4 and DN T cells. A) Heat map and hierarchical cluster analysis of genes that were differentially regulated over 4 fold are depicted for DN (lane 1), CD4 (lane 2) and CD8 T cells (lane 3). Red indicates upregulation and green indicates down regulation for each gene listed on the right. B) Venn Diagram representing overlap of differentially regulated genes (2 fold) identified from the microarray analysis of CD4, CD8 and DN T cells stimulated through their TCR (anti CD3/CD28).

Gene expression analysis of purified CD4, CD8 and DN T cells from uninfected mangabeys were analyzed by both array and real-time PCR analysis. Microarray analysis undertaken to evaluate TCR stimulation (anti-CD3/anti-CD28) determined that CD4 T cells differentially regulated (over 2 fold with p<0.05) 2005 genes, whereas CD8 and DN T cells differentially regulated fewer genes, 1254 genes and 1123 genes respectively. Some of the immunomodulatory genes are depicted in the heat-map (Figure 2A) highlighting the impact of the TCR stimulation on DN T cells (lane 1), CD4 T cells (lane 2) and CD8 T cells (lane 3). Among the 122 genes differentially regulated in each cell type, there were numerous immune modulatory transcripts including cytokines and chemokines (IFNγ, CCL4L1 (CCR5 agonist) and XCL1) as well as transcription factors (RGS8 and BATF). The similarity between DN, CD4 and CD8 T cells can be visualized via a Venn diagram (Figure 2B), which depicts 509 genes regulated to the same extent in DN and CD4 T cells and 248 genes regulated similarly between DN and CD8 T cells. Additionally, a hierarchical cluster analysis (Pearson's algorithm) of highly upregulated genes (over 4 fold with p<0.05) determined that genes upregulated by DN T cells more closely cluster with CD4 T cells rather than CD8 T cells (Figure 2A, phylogenetic tree above the heat map). Genes differentially regulated similarly in the DN and CD8 T cells subsets were often involved in ion channel and signaling function (KCNK5, RIPK2 and ERG2) as well as the costimulatory molecules such as ICOS. Interestingly, many of the differentially regulated genes shared by CD4 and DN T cells were associated with T helper function including IL4, IL17F, IL22 and IL2RA; chemokines such as CXCL9, CXCL10 and CXCL11 and other immune genes such as MIP3α (CCL20) and IRF8 (Figure 2)

**Figure 3:**
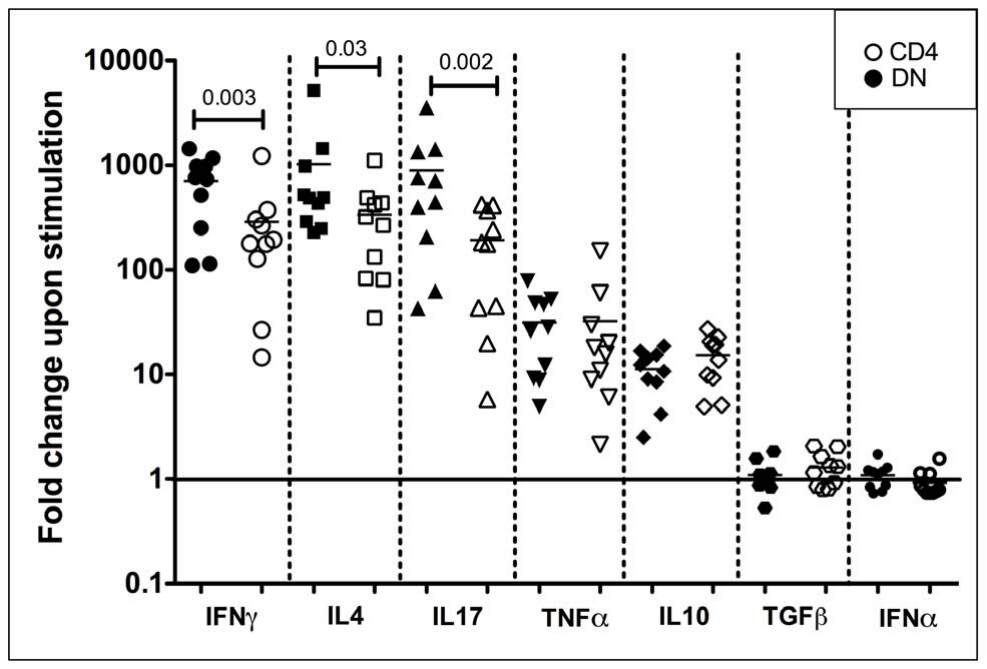
Quantitative real time PCR analysis of TCR stimulated DN and CD4 T cells from uninfected mangabeys. Purified DN (filled symbols) and CD4 (open symbols) T cells isolated from ten uninfected magabeys were stimulated with anti-CD3/anti-CD28. Upon stimulation, DN T cells upregulate IFNγ, IL4 and IL17 mRNA to the greatest extent with higher expression of these genes (paired analysis) in DN compared to CD4 T cells. TNFα and IL10 mRNA were also upregulated by both DN and CD4 T cells. TGFβ and IFNα expression was not altered following TCR stimulation in either DN or CD4 T cells. Log scale fold change is shown on the Y-axis with no change in mRNA expression due to stimulation indicated by a baseline (1 fold). Symbols represent IFNγ (large circle), IL4 (square), IL17 (triangle), TNFα (inverted triangle), IL10 (diamond), TGFβ (hexagon) and IFNα (small circle).

In order to quantify and compare these shared T helper cytokines between CD4 and DN T cells, we performed qPCR analysis on purified CD4 (open symbols) and DN T cells (filled symbols) from 10 uninfected mangabeys focusing on seven key cytokines. These cytokines included those specific for Th1 (IFNγ), Th2 (IL4), Th17 (IL17), pro-inflammatory (TNFα), anti-inflammatory (IL10), Treg (TGFβ) and antiviral (IFNα) cytokines. Following stimulation through the T cell receptor (anti-CD3/CD28) DN T cells exhibited the strongest upregulation in three canonical T helper cytokines IFNγ (700 fold, Th1), IL4 (1000 fold, Th2) and IL17 (900 fold, Th17), with slightly less upregulation for the pro-inflammatory cytokines TNFα (30 fold) and anti-inflammatory IL10 (10 fold) (Figure 3, filled symbols). No upregulation was observed in TGFβ (Treg) or IFNα (antiviral) following TCR stimulation, although DN T cells did express these cytokines at a basal level. Similarities were clear in the pattern of cytokine expression between DN and CD4 T cells (Figure 3), although DN T cells demonstrated a greater upregulation of IFNγ (2.4 fold, p = 0.003), IL4 (3 fold, p = 0.03) and IL17 (5 fold, p = 0.002) than CD4 T cells (Figure 3). Stronger mitogenic stimulus with PMA/Ionomycin also revealed a similar functional profile in DN and CD4 T cells (Figure S4); however, the significant differences between CD4 and DN T cells were no longer evident, indicating that DN T cells respond in a more similar manner to CD4 T cells when provided a strong stimulus.

### Transcriptome analysis of DN T cells from SIV+ mangabeys identified a maintained T-helper function

**Figure 4:**
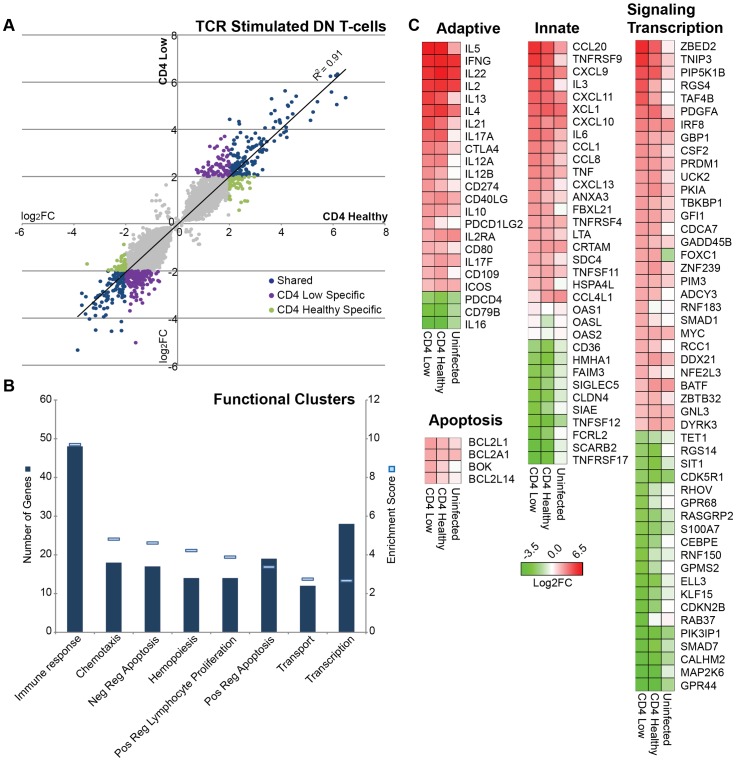
Transcriptome analysis utilizing microarray of TCR stimulated DN T cells from mangabeys depicts upregulation of transcription factors and immunomodulatory genes. Microarray analysis comparing TCR-stimulated and unstimulated DN T cells. A) DN T cells from two SIV+CD4-healthy and two SIV+CD4-low mangabeys were stimulated with anti-CD3/anti-CD28 and comparative mRNA expression of differentially regulated genes following stimulation of is depicted with less than 4 fold differential regulation depicted in grey, while those with greater than 4 fold differential expression depicted in color (n = 568). In blue are genes regulated to the same extent in DN T cells from both SIV+CD4-low and SIV+CD4-healthy mangabeys, while genes specific to DN T cells from SIV+CD4-low animals are depicted in purple and those specific to DN T cells from SIV+CD4-healthy animals are in green. B) Enrichment data of functional clusters in gene families that are differentially regulated upon stimulation with number of genes shown as columns (dark blue) and extent of enrichment of functional gene clusters (enrichment score) depicted as lines (light blue). Genes involved in immune response and transcription were highly enriched. C) Evaluation of immunomodulatory genes with subsets representing adaptive immunity, innate immunity, apoptosis, signaling and transcription genes are presented as a heat map. Differentially regulated genes from SIV+ CD4-healthy, SIV+ CD4-low and uninfected mangabeys with ≥4 fold differential regulation following TCR stimulation depicting upregulated (red) and downregulated (green) gene expression.

We next analyzed whether SIV infection or SIV-induced CD4 T cell loss impacts the functional potential of DN T cells. Microarray profiles of DN T cells were evaluated from 2 SIV+ CD4-low, 2 SIV+ CD4-healthy and 2 uninfected mangabeys. Following TCR-stimulation (anti-CD3/CD28), DN T cells differentially regulated 263 genes over 4 fold (p<0.05) in the SIV+ CD4-low and SIV+ CD4-healthy animals compared to unstimulated DN T cells (Figure 4A, Blue circles). Several genes were also differentially regulated only in the CD4-low (Figure 4A, Purple circles n = 215) or CD4-healthy mangabeys (Figure 4A, green circles, n = 90). Interestingly, SIV+ CD4-low mangabeys exhibited a greater number of genes that are either up- or down-regulated when compared to SIV+ CD4-healthy mangabeys (478 versus 353 respectively) indicating that the function of DN T cells may be altered following a prolonged depletion of CD4 T cells (Figure 4A).

Differentially regulated genes were assessed for enrichment of Gene Ontologies using the DAVID Bioinformatics Database [44], [45], and genes with related ontologies clustered into functional groups. Based on this unbiased assessment, upregulation of immune response genes was the most prominent among the ontologies (enrichment for immune genes) in addition to a number of more specific functional ontologies, including chemotaxis, apoptosis, T-cell proliferation and transcription (Figure 4B). Guided by these ontologies, we further classified the data, using heat maps to represent differential expression upon TCR stimulation, separating the immune response ontology into innate and adaptive responses, and generating a broad category of immune modulatory signaling and transcription. From several hundred differentially regulated genes, 111 genes with known immune modulatory function are presented (Figure 4C). Transcription factors and signaling molecules are the largest group of genes upregulated by DN T cells after TCR stimulation with over 4 fold upregulation in ZBED2, TNIP3, RGS4, IRF8, GADD45B and SMAD1 (Figure 4C). Interestingly, a number of genes, including signaling molecules such as RGS4 and TNIP3, were upregulated after SIV infection and differentially upregulated between CD4-low and CD4-healthy mangabeys (5 and 2 fold higher respectively; Table 2). However, some transcription factors such as SMAD7 were downregulated in the DN T cells particularly in the CD4-low mangabeys.

**Table 2:** Differentially regulated genes between SIV+ CD4-low and SIV+ CD4-healthy mangabeys.

Fold change – Higher in CD4-low				Fold change – Higher in CD4-Healthy			
Gene	CD4 Healthy	CD4 Low	Ratio L/H	Gene	CD4 Healthy	CD4 Low	Ratio H/L
Upregulated genes				Upregulated genes			
RGS4	4.0	19.6	4.8	TWIST1	9.5	4.3	2.2
TAF4B	5.6	18.7	3.3	DDX21	6.9	4.3	1.6
CPXM1	5.7	17.8	3.1	IL2RA	7.6	4.7	1.6
TSLP	4.7	12.5	2.7	IL22	42	28.5	1.5
NETO2	5.3	13.4	2.5	Downregulated genes			
FRMD4B	5.9	14.9	2.5	ARHGAP6	7.2	4.1	1.8
SLC16A1	4.4	10.0	2.3	ANKRD58	13.6	8.7	1.6
TNIP3	13.2	28.0	2.1	KLHL3	8.9	6.0	1.5
IL12B	4.4	8.0	1.8				
ZBED2	17.2	31.7	1.8				
GBP1	4.6	8.3	1.8				
IL21	13.0	21.7	1.7				
SLC26A4	5.0	8.3	1.7				
RIPK2	9.0	14.7	1.6				
PRRX2	7.5	12.2	1.6				
CXCL10	8.1	13.0	1.6				
Downregulated genes							
SMAD7	4.2	9.5	2.3				
PSRC1	7.6	15.0	2.0				
NOG	14.9	28.7	1.9				
PIK3IP1	4.7	8.6	1.8				
ERP27	4.6	8.2	1.8				
SNPH	6.4	11.3	1.8				
ROM1	5.0	8.5	1.7				
ARAP3	4.9	8.1	1.6				
CSPG5	8.1	13.0	1.6				
FRMD3	4.4	7.0	1.6				

**Figure 5:**
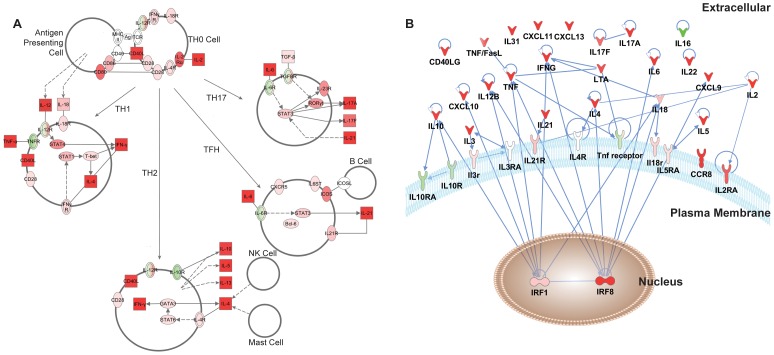
Network interactions of differentially regulated genes in DN T cells. A) An unsupervised *in silico* model of genes upregulated in DN T-cells upon TCR stimulation identifies cytokines characteristic of T-helper differentiation (Th1, Th2, T_FH_ and Th17 subtypes) with upregulated (red) and downregulated (green) gene expression depicted. The model also depicts how cytokines expressed by DN T cells may influence immune cells such as antigen presenting cells, B cells, NK cells and mast cells. Squares represent secreted proteins whereas ovals depict receptors or regulatory proteins. B) A network of interactions between the most highly regulated cytokines and their respective regulatory proteins from TCR stimulated DN T cells. Nodes (gene names) represent the upregulated (red) and downregulated (green) cytokines. Edges (blue lines) represent direct interactions between the cognate proteins. The compartmentalization of these proteins into extracellular expression, plasma membrane surface expression and nuclear expression is also depicted.

Strong upregulation was observed for genes associated with classical Th1 function in both CD4-low and CD4-healthy mangabeys, including the signature cytokine IFNγ as well as interferon stimulated chemokines CXCL9, CXCL10 and CXCL11 (with the highest CXCL10 expression in DN T cells from CD4-low mangabeys, Table 2). This chemokine expression signature was maintained in DN T cells from both uninfected and SIV infected mangabeys (Figure 4C). DN T cells also have a potential for Th2 function as indicated by the upregulation of CD40LG as well as cytokines IL4, IL5, IL13, and IL10. CD40LG, IL4 and IL10 expression was maintained irrespective of SIV infection although the expression of IL5 and IL13 was increased most dramatically in DN T cells from SIV infected mangabeys (Figure 4C). Cytokine mRNAs associated with canonical Th17 function IL17, IL6, TNFα, IL22 were maintained after SIV infection and exhibited a greater upregulated in CD4-low compared to CD4-healthy mangabeys (Table 2). T_FH_ (T follicular helper) function (IL21, ICOS, CD40L, IL6) was assessed in DN from infected and uninfected mangabeys. Although ICOS, CD40L and IL6 function remained similar irrespective of SIV infection, IL21 was strongly induced in the DN T cells from SIV infected mangabeys (Figure 4C). Interestingly, an unsupervised *in silico* pathway analysis based on all significantly upregulated genes also indicated with a high degree of confidence (p = 5.94E^−12^) that the expression patterns of DN T cells is consistent with helper T-cell attributes and include Th1, Th2, Th17 and T_FH_ functionality (Figure 5A).

Gene expression data were integrated with extant literature to generate a network of direct interactions between key cytokines, chemokines and related molecules, revealing interesting regulatory features (Figure 5B). For example, the expression of IL2 as well as its cognate receptor IL2RA and the enrichment of the lymphocyte proliferation ontology points to an ability of DN T cells to proliferate in response to this T cell survival cytokine. Conversely, although the pro-inflammatory cytokine TNFα and anti-inflammatory cytokine IL10 were upregulated, their specific receptors, IL10R, IL10RA and TNF receptor were downregulated in the DN T cells, indicating that DN T cells buffer their own pro- and anti-inflammatory capacity, yet aid in providing a cytokine milieu to potentiate immune responses upon TCR stimulation. (Figure 5B). Taken together, these expression data and systems-level analyses demonstrate that DN T cells from both CD4-low and CD4-healthy SIV infected mangabeys signal through their T cell receptors in the absence of CD4 and CD8 and express cytokines/chemokines associated with Th1, Th2, T_FH_ and Th17 helper T cells.

### CD4-like functionality of DN T cells is maintained irrespective of virally mediated CD4 T cell loss

**Figure 6:**
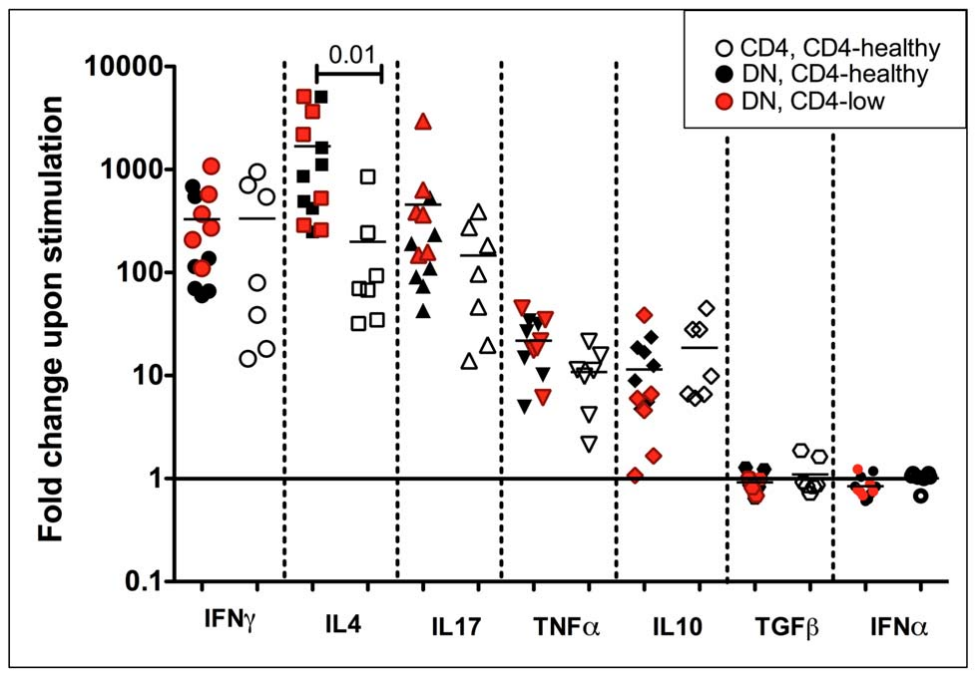
Quantitative real time PCR analysis of TCR stimulated DN and CD4 T cells from SIV infected mangabeys. Real time PCR analysis of purified DN and CD4 T cells isolated from SIV+ mangabeys demonstrates upregulation of IFNγ, IL4, IL17, TNFα and IL10 upon TCR-stimulation. Cytokine expression of DN T cells from SIV+ CD4-healthy mangabeys (black symbols), SIV+ CD4-low mangabeys (red symbols) and CD4 T cells from SIV+ CD4-healthy mangabeys (open symbols) are depicted. Cytokine profiles of DN T cells are maintained irrespective of CD4 T cell loss. Log scale fold change is shown on the Y-axis with no change in mRNA expression due to stimulation indicated by a baseline (1 fold). Symbols represent IFNγ (large circle), IL4 (square), IL17 (triangle), TNFα (inverted triangle), IL10 (diamond), TGFβ (hexagon) and IFNα (small circle).

The ability of DN T cells to compensate for CD4+ T cell loss during SIV infection can be further inferred by evaluating DN from SIV+ CD4-healthy (n = 7) and SIV+ CD4-low mangabeys (n = 6) to the functional profile to CD4 T cells from the CD4-healthy mangabeys (SIV+ CD4-low mangabeys had too few CD4 T cells to isolate and analyze in this manner). Similar to our evaluation of cells from uninfected mangabeys (Figure 3), cytokine transcripts associated with Th1 (IFNγ), Th2 (IL4), Th17 (IL17), pro-inflammatory (TNFα), anti-inflammatory (IL10), Treg (TGFβ) and antiviral (IFNα) were evaluated (utilizing quantitative real-time PCR) following stimulation through the T cell receptor (anti-CD3/CD28 – Figure 6) or through a mitogenic stimulus (PMA/Ionomycin – Figure S5). A paired evaluation of the mRNA expression of these cytokines following TCR stimulation in DN and CD4 T cell was undertaken in SIV+ CD4-healthy mangabeys. The mean upregulation of the cytokines IFNγ (500 fold), IL17 (800 fold), IL10 (20 fold) and TNFα (50 fold) was similar in the DN and CD4+ T cells (Figure 4, black symbols compared to open symbols). The one difference observed was that IL4 upregulation was higher in the DN compared to the CD4 T cells, indicating that DN T cells were particularly adept at making this cytokine (Figure 4). Following a more robust stimulation with the mitogen PMA/Ionomycin, a significant elevation in cytokine mRNA levels (compared to TCR stimulation) was observed in both DN and CD4 T cells however no significant difference in was observed between CD4 and DN T cells (Figure S5). IFNα and TGFβ mRNA were detectable, but their levels were not upregulated in DN or CD4 T cells following either TCR or mitogen stimulation. Importantly, we observed that DN T cell function was similar in both SIV+ CD4-low mangabeys (Figure 6, red symbols) and SIV+ CD4-healthy mangabeys (black filled symbols) following either TCR or PMA/Ionomycin stimulation (Figures 6 and S5). There was no significant difference between cytokine mRNA expression of DN T cells between CD4-low and CD4-healthy mangabeys in these signature T helper cytokines indicating a maintained function despite CD4 loss.

### SIV infection impacts IFNγ protein levels in DN T cells and can be inhibited by both MHC-I and MHC-II antibodies

**Figure 7:**
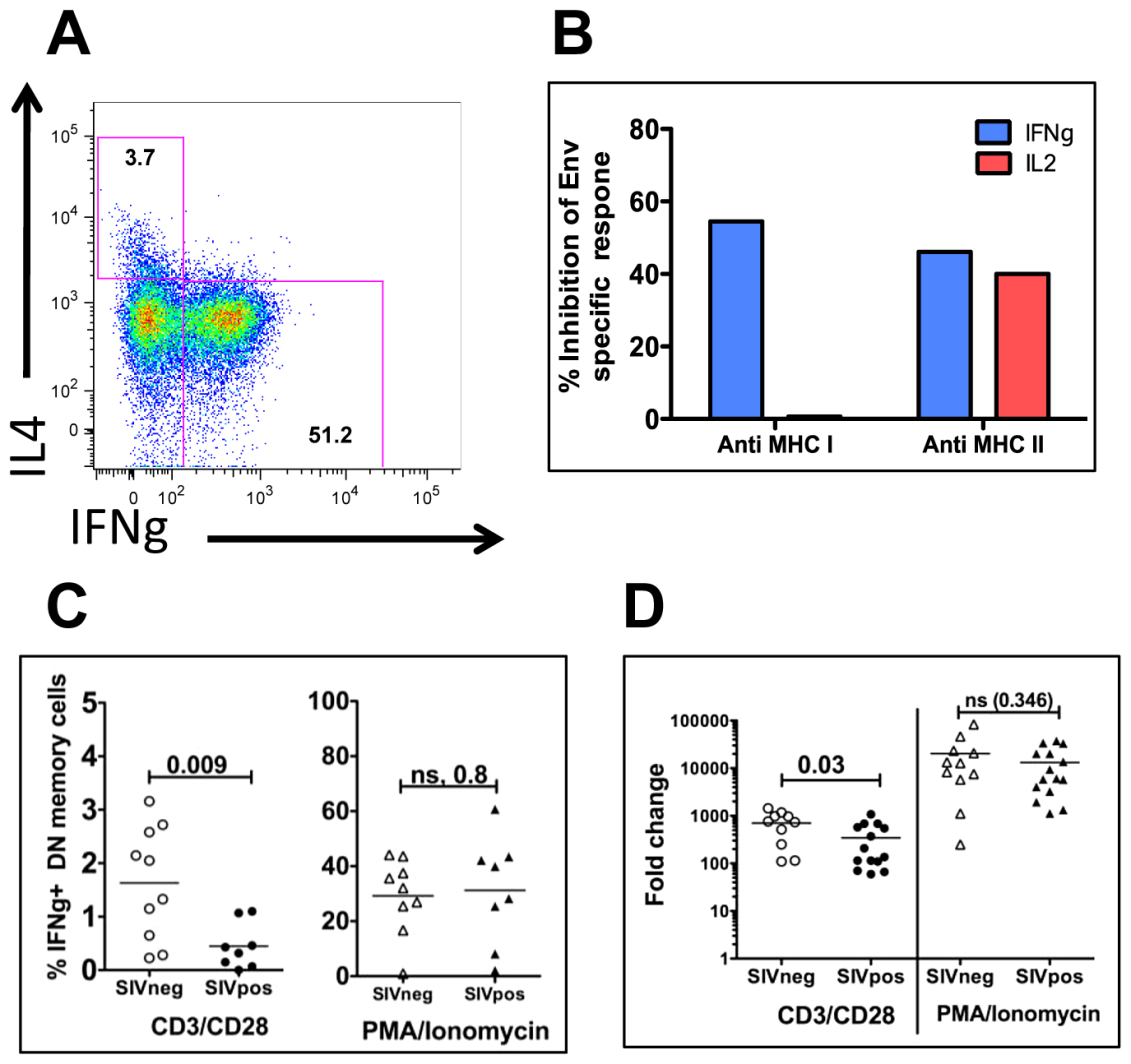
Cytokine expression (flow cytometric and quantitative real-time PCR) and MHC restricted response of DN T cells. A) Representative flow cytometry plot of IFNγ protein expression (x-axis) and IL4 protein expression (y-axis) indicating that different DN subpopulations generally express either IFNγ or IL4 (not both). B) Quantification of the percent inhibition of SIV-Env peptide specific responses within DN T cells in the presence of MHC I (left side) or MHC II (right side) specific antibodies. Intracellular cytokine staining identifies IFNγ (blue) or IL2 (red) expressed by DN T cells within PBMC of a SIV infected mangabey. There was minimal inhibition of IL2 expression in the presence of MHC-I antibody. C) Flow cytometric analysis of mangabey PBMCs, indicating that TCR stimulation results in a greater percentage of DN T cells expressing IFNγ protein in uninfected compared to SIV+ mangabeys. In contrast, mitogenic stimulation (PMA/Ionomycin) did not result in any difference in IFNγ expression between these two groups. D) IFNγ mRNA expression indicating an increased expression of IFNγ mRNA from DN T cells from uninfected mangabeys upon stimulation through their TCR, but not when stimulated with PMA/Ionomycin.

Differential expression of cytokines in the SIV infected and uninfected mangabeys have the potential to uncover key insights into DN T cell function. Comparison of IFNγ expression by DN T cells in SIV infected and uninfected mangabeys identified a two-fold higher upregulation of IFNγ in the uninfected mangabeys following CD3/CD28 TCR stimulation (Figure 7). This difference was statistically significant at both the protein (1.7% in uninfected and 0.7% in infected - Figure 7C) and mRNA (700 fold in uninfected and 350 fold in infected - Figure 7D) levels. This difference was not observed following a stronger mitogenic PMA/Ionomycin stimulation (Figure 7D, S4, S5). Further, intracellular cytokine production analysis revealed that DN T cells expressed either a Th1 (IFNγ) or Th2 (IL4) cytokine profile (Figure 7A) [46], [47]. These findings provide evidence that, like CD4 T cells, DN T cells exist as different functional subpopulations and that SIV infection impairs the ability of these cells to produce IFNγ, a cytokine that has the ability to direct the immune system primarily toward a Th1 response. No differences were evident between infected and uninfected mangabeys in any of the other cytokine mRNA measured by qPCR or cytokine protein levels measured by intracellular cytokine staining.

We also assessed the ability of DN T cells to respond to SIV Env specific peptides in the context of MHC binding. Blocking experiments with anti-MHCI or anti-MHC-II antibodies demonstrated that IFNγ secretion by DN T cells is mediated through either MHC-I (54% inhibition) or MHC-II (46% inhibition) dependent pathways. In contrast, Env peptide stimulation of IL2 secretion was mediated primarily through an MHC-II dependent manner (40% inhibition) and was not inhibited by MHC-I antibodies (Figure 7B). Taken together, these data demonstrate that with regard to IFNγ secretion, DN T cells can recognize antigens presented by either MHC-I or MHC-II molecules. However, DN T cells the express the proliferation cytokine IL2 are driven primarily through a MHC-II mechanism. These data may indicate that one DN T cell can respond to both MHC types, or rather that one population responds to antigen presented by MHC-I and a different DN subpopulation responds to antigens presented by MHC-II.

### DN T cells maintain proliferative capacity upon SIV infection

**Figure 8:**
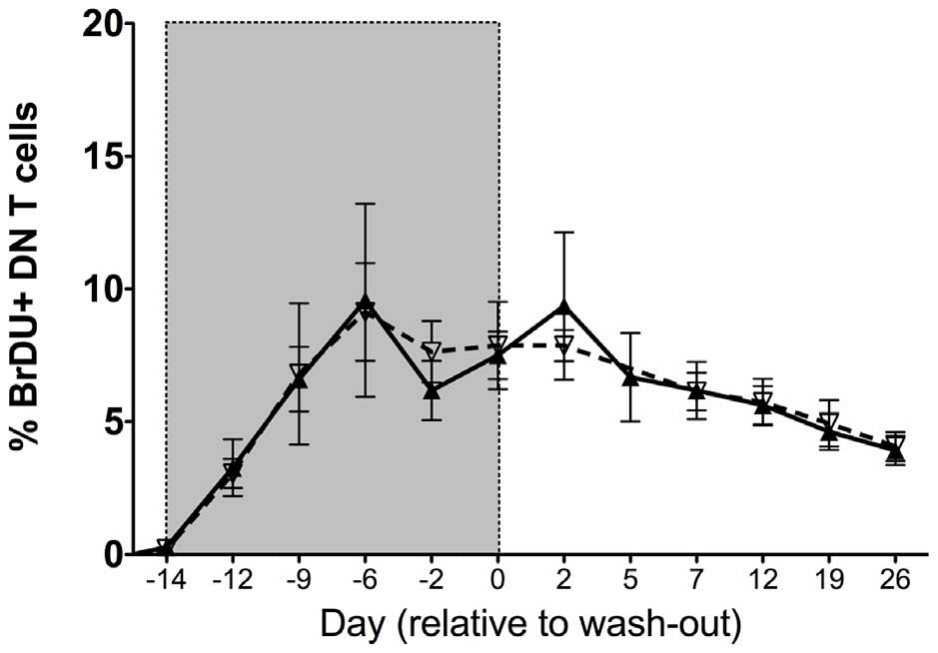
In vivo evaluation of proliferation and turnover of DN T cells in mangabeys. 5-Bromo-deoxyUridine (BrDU) labeling was undertaken in 3 uninfected (dotted line, mean) and 5 chronically SIV infected mangabeys (solid line, mean). Analysis of PBMCs indicates that DN T cells incorporate BrDU during the labeling phase (gray shaded area) at a similar rate in the SIV infected and uninfected mangabeys. During the wash-out phase (not shaded) the turnover rate implied by the loss of BrDU from cells was also similar between SIV infected and uninfected mangabeys. Mean ± SEM depicted for each time point.

**Figure 9:**
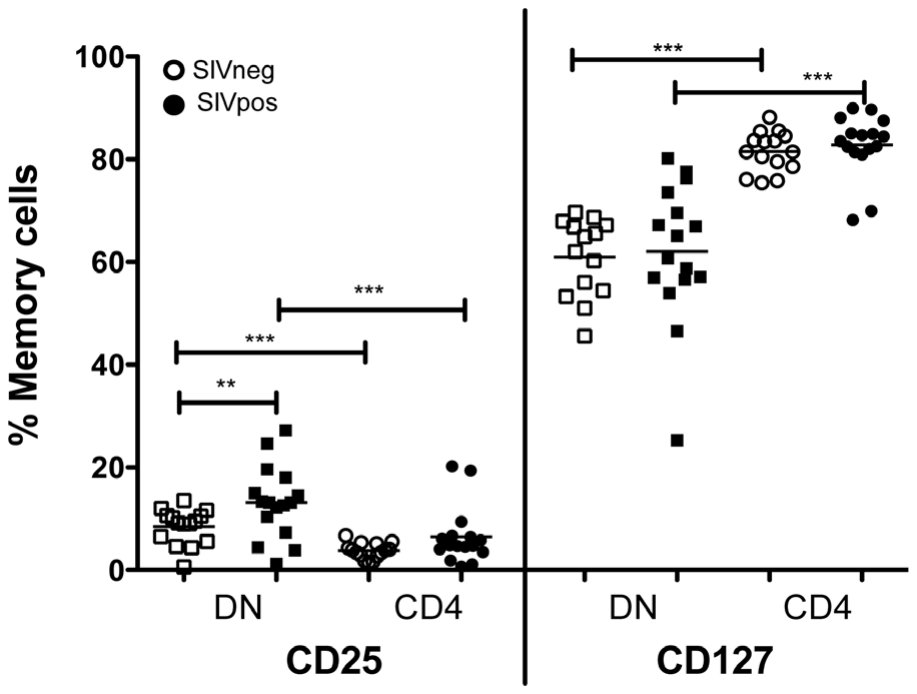
Flow cytometric analysis of homeostatic cytokine receptor expression of DN and CD4 T cells. Expression of IL2 receptor (CD25) and IL7 receptor (CD127) was assessed in memory population (CD95+) of DN and CD4 T cells from 15 uninfected (open symbols) and 15 SIV infected (filled symbols) mangabeys. CD25 expression was elevated in DN T cells from SIV infected mangabeys compared to uninfected mangabeys. In addition, CD25 expression was higher in DN T cells compared to CD4 T cells in a paired comparison. CD127 expression was higher in CD4 in comparison to DN T cells and these levels were not impacted following SIV infection.

To evaluate DN T cell proliferation and turnover, 3 uninfected and 5 chronically SIV infected mangabeys were treated with the thymidine analogue 5-bromo-2′-deoxyuridine (BrdU) for 14 days (Figure 8). BrdU incorporation was monitored throughout the labeling period and for a period of 90 days following treatment. The BrdU uptake by the DN T cells during labeling period and the kinetics of BrdU during the wash-out was similar in both SIV infected and uninfected mangabeys (Figure 8). This finding was further verified through expression of the Ki67 protein by flow cytometry, which was similar in the mangabey DN T cells irrespective of their infection status (data not shown). In addition, we assessed the expression of the IL2 receptor (CD25) and IL7 receptor (CD127) on DN T cells from SIV infected and uninfected mangabeys to evaluate their potential to respond to the homeostatic cytokines (Figure 9). CD25 was expressed on 8% of DN T cells from uninfected mangabeys and at a significantly higher level (14%) in SIV infected mangabeys. Additionally, a higher percentage of DN T cells expressed CD25 compared to CD4 T cells in both infected and uninfected mangabeys (Figure 9). CD127 was consistently expressed at a high level in DN T cells from both SIV infected and uninfected mangabeys with a mean expression of 62% (Figure 9), albeit at lower levels than CD4 T cells (mean expression 81%). Taken together these data show that mangabey DN T cells proliferate during SIV infection and express levels of homeostatic cytokine receptors that demonstrate a potential for responding to both IL2 and IL7.

## Discussion

This study demonstrates that (CD3+CD4−CD8−) DN T cells possess Th1, Th2, Th17 and T_FH_ function in sooty mangabeys irrespective of SIV infection. We expand upon previous findings by performing an in-depth analysis of DN T cell functions using transcriptomic as well as flow cytometric analyses, and now define how these functions are altered during SIV infection and SIV mediated CD4 T cell loss. DN T cells are refractory to SIV-infection [34], which likely aids their ability to maintain function and proliferative capacity during SIV infection and the subsequent CD4 depletion observed in the CD4-low mangabeys [11], [15], [16]. We identify DN T cells as a polyclonal T cell subset with a predominantly EM cell phenotype, maintained proliferative capacity, and with a specific increase in the proportion of EM DN T cells. Further, we show that they have the ability to respond to SIV specific antigens presented in the context of both MHC-I or MHC-II. Previous studies have demonstrated that during SIV infection of mangabeys, CD4 T cells retain a CM phenotype and this preservation of CM T cells has been implicated as one of the mechanisms by which mangabeys are able to inhibit progression to simian AIDS [13]. Since EM CD4 T cells are infected at greater frequency than CM CD4 T cells in the sooty mangabeys, the DN T cells, which are refractory to SIV infection, may therefore perform T helper effector functions and serve as workhorses for the mangabey immune system, with a potential to function during SIV infection.

Whole transcriptome microarray analysis was previously utilized in non-sorted peripheral blood cells from natural hosts to demonstrate that natural hosts can produce robust innate immune responses and upregulate interferon stimulated genes (ISGs) following SIV infection [48]. Our transcriptome analysis of DN T cells demonstrated that TCR stimulation results in an upregulation of numerous transcription factors, signaling proteins and immune modulatory proteins despite the lack of CD4 or CD8 proteins. When CD4 and CD8 T cells respond to antigens in the context of either MHCII or MHC I respectively, the protein tyrosine kinase Lck (p56) associated with the intracellular domains of CD4 or CD8 is responsible for initiating the signal transduction through the TCR [49], [50]. One possibility for DN signaling in the absence of these critical molecules is that DN T cells are less dependent on Lck signaling [27], [51], [52] and activation of Fyn along with LAT and Erk kinases may be sufficient to activate ITAMs in DN T cells [52]–[54]. Our analysis has shown that DN T cells do respond to SIV specific peptides in both a MHC I and MHC II dependent manner (through production of IFNγ). We hypothesize that DN T cells are able to respond to antigens presented by either MHC molecule due to the absence of either a CD4 or CD8 protein in these cells.

IFNγ is a Th1 cytokine that was highly expressed in TCR-stimulated DN T cells (500 fold upregulation by real-time PCR). IFNγ plays a role in clearance of intracellular pathogens by macrophages and is critical for antigen specific T cell responses. IFNγ stimulated genes CXCL9, CXCL10 and CXCL11 were also strong upregulated and maintained in the DN T cells irrespective of SIV infection or SIV-induced CD4 T cell loss. The strong upregulation of IFNγ by DN T cells provides evidence that they can influence both innate and adaptive immune response by enhancing macrophage antimicrobial activity and CD8 T cell function. Here we demonstrate that SIV infection is associated with a decrease in IFNγmRNA and protein expression in the DN T cells (Figure 7C,D). We hypothesize that this SIV related IFNγ down-modulation may play a role in the suppressed immune activation observed during the chronic phase of the infection in mangabeys compared to SIV infected macaques and HIV-infected humans.

A functional humoral immune response requires upregulation of T_FH_ (follicular helper) and Th2 cytokine expression in order to provide B cell help. Unsupervised *in silico* analysis (Figure 5) of DN cytokine expression indicated that DN T cells possess Th2 function, as evidenced by upregulation of signature cytokines IL4, IL5, IL10 and IL13 (Figure 2A, 4C) necessary for activation of antigen-specific B cells and the production of antibodies. It is interesting that SIV infection augments the ability of DN T cells to upregulate IL5 and IL13 while maintaining IL4 and IL10 levels (Figure 4C). In addition, our analysis also indicates potential T_FH_ (T follicular helper) function in DN T cells. Peripheral blood T_FH_ cells generally have a memory phenotype [55], [56], express the cytokine IL21, and express high levels of CD40 ligand (CD40L) as well as inducible costimulator (ICOS) required for B cell activation. They also secrete IL6, augmenting IL21 production to assist in generating antibody responses [57]. Transcriptome analysis (Figure 2, 4) determined that DN T cells in SIV infected mangabeys upregulate IL21, ICOS, CD40L and IL6 indicating the potential for T_FH_ functionality. This pattern of IL4, IL5, IL10 and IL13 expression in addition to IL21, ICOS and CD40L suggests that DN T cells can provide B cell help to maintain adaptive humoral responses when CD4 levels are low due to SIV infection. Indeed, SIV+ CD4-low mangabeys are able to generate recall antibody responses to vaccination against Influenza [16] and DN T cells have the potential to assist in facilitating this helper T cell function.

Preservation of Th17 function in natural hosts is critical to a non-pathogenic disease course following SIV infection, as Th17 T cells are involved in maintaining gut mucosa integrity [58]–[61]. Th17 cells are selectively depleted during pathogenic SIV infection of macaques and in HIV+ humans [14], [58], [59]. Th17 function in DN T cells was demonstrated by the upregulation of Th17 cytokines (IL17 and IL22 [60]–[62]) as determined by both arrays and real-time PCR analysis indicate that this Th17 function was maintained in DN T cells irrespective of SIV infection. The maintenance of Th17 function by DN T cells during SIV-infection may allow the natural host immune system access to the Th17 cytokines critical for maintaining mucosal integrity to prevent microbial translocation that would otherwise contribute to chronic immune activation.

A key feature of SIV infected natural hosts in limiting disease progression is their ability to limit chronic phase immune activation, the mechanism for which is still under investigation [3], [5], [10], [63]. The cytokine profile of maintained Th17 function, reduced IFNγ expression upon SIV infection (discussed above), and upregulation of the classic anti-inflammatory cytokine IL10 by DN T cells may contribute towards controlling this systemic immune activation. Additionally DN T cells also strongly express the Th17 cytokine IL22 (Figure 2 and 4), which can have anti-inflammatory properties when acting in the presence of with IL10 [64]. Interestingly, IL10 upregulation in DN T cells occurs concurrently with a down regulation of IL10 receptors – IL10R, IL10RA, raising the possibility that the DN T cells suppress inflammation in other cells, while inhibiting their own response to IL10. This may allow DN T cells to remain fully activated and functional while contributing to lower immune activation in the mangabeys. Recent studies have demonstrated that in primary HIV infection there is a strong correlation between higher DN T cell levels early in infection and lower HLADR+CD38+ CD8 T cells later in infection [30]. This reduced immune activation was associated with the expression of anti-inflammatory cytokines IL10 and TGFβ by DN T cell subset that does not express FoxP3 [30] (a marker for Treg function in CD4 T cells). Previous studies of DN T cells in sooty mangabeys demonstrate that DN and CD4 T cells express similar levels of FoxP3 [34]. Our findings also demonstrate the expression of FoxP3 by DN T cells, although no upregulation was detected after TCR stimulation. Our transcriptomic analysis of TCR stimulated DN T cells also identified a strong upregulation in transcription factor TNIP3 (TNFAIP interacting protein 3) an anti-inflammatory gene that limits NF-κB signaling and signaling molecule RGS4 (also a negative regulator [65]), thus contributing to control of excessive/sustained immune activation during chronic SIV infection. This functional profile of DN T cells in mangabeys, characterized by higher DN T cell number and IL10 expression supports our hypothesis that DN T cells aid in the control of immune activation during the chronic phase of SIV infection of natural hosts. These multi-functional roles of DN T cells suggest that DN T cells are functional (including their anti-inflammatory role) irrespective of CD4-T cell levels or SIV infection status. It is therefore likely, that similar to CD4 T cells, a subpopulation of DN T cells has a regulatory function.

These findings characterize DN T cell functionality in sooty mangabeys, providing evidence for DN T cell subset function that is similar to CD4 T helper cells, and a maintained ability of these cells to proliferate and function following the SIV infection. As a population recalcitrant to SIV infection, DN T cells have the potential to contribute to the maintenance of an effective immune and may be evolution's answer to preserve helper T cell functions during a CD4 T cell depleting infection. We propose that DN T are a candidate immune therapeutic target to decrease disease progression in HIV+ patients. One approach to increase DN T cell functional potential would be to increase their levels, as they exist at a higher frequency in non-pathogenic hosts compared to humans [34], One candidate therapy is to promote the expansion of the DN T cell populations is interleukin 7 (IL7), a cytokine involved in the homeostasis and maintenance of T cell populations [66], [67]. The presence of the IL7 receptor (CD127) on over 60% of DN T cells even during SIV infection (Figure 8) suggests that these cells have the potential to respond to IL7 therapy. Several clinical trials utlizing IL7 therapy in HIV-infected patients and SIV infected Rhesus macaques have demonstrated promising increases in both CD4+ and CD8+ T cell pools in the peripheral circulation [66]–[72], including the naïve and central memory CD4+ T cell compartments [66], [73]. A second approach could be expanding function of DN T cells from pathogenic hosts to encompass Th1, Th2, Th17, and T_FH_ functions (as observed in SIV+ mangabeys). Analysis of DN T cell function in the humans have generally focused on the regulatory potential [28], [29] of these cells and other specific functions are currently being investigated. These studies indicate the potential for utilizing DN T cells as future immune therapeutic targets (potentially utilizing IL7, IL2 or a combination of IL2/IL7 therapy) to increase their levels and/or function during pathogenic HIV infection as one component of a functional cure to inhibit progression to clinical disease and AIDS.

## Materials and Methods

### Ethics statement

All animals involved were cared for in accordance with NIH guidelines as well as by approved protocols with the Seattle Biomedical Research Institute's Institutional Animal Care and Use committee (IACUC) committee (#DS-NHP-Yerkes) and Yerkes National Primate Research Center's IACUC approved protocol (IACUC #2000280). Appropriate measures were taken to assure that discomfort, distress, pain and injury was limited to that which is unavoidable in the conduct of the research plan. Ketamine (10 mg/kg) and/or Telazol (4 mg/kg) were used for sedation and analgesics were used when appropriate as determined by the veterinary medical staff.

### Animals

We obtained mangabey blood from colony bred sooty mangabeys (*Cercocebys atys*) at the Yerkes National Primate Research Center. The SIV infected mangabeys utilized in this study were either naturally infected in the Yerkes colony or experimentally infected as previously described [16]. Five SIV infected CD4-low mangabeys are part of the previously published Sodora Lab study [16] and we have also included one mangabey present within the naturally SIV infected group within the Yerkes colony that also had a CD4-low phenotype.

### Flow cytometry

All antibodies were obtained from BD unless specified. Peripheral blood mononuclear cells (PBMC) were isolated from blood of SIV infected and uninfected mangabeys via Ficoll-Hypaque gradient. PBMCs were stained with CD3 (SP34 clone) APC-Cy7 or Alexa 700; CD4 (L200) Pacific Blue or PerCP-Cy5.5; CD8 (SK1 clone) PerCP-Cy5.5 or (RPA-T8 clone) Pacific Blue; CD28 (28.2 clone) PE-Cy7 or FITC, CD95 (DX2 clone) PE-Cy5 or APC, CD62L PE to perform identify naïve and memory phenotypes. Analysis of proliferating cells were performed using Ki67 (B56 clone) FITC and BrDU (3D4) APC along with CD8 (SK11) APC-Cy7, CD3 (SP34-2 clone) Alexa 700 and CD4 (OKT4 clone) Pacific Blue. Cytokine receptor analysis utilized CD25 PE-Cy7 and CD127 PE antibodies. IFNγ (4S.B3 clone) PE and IL4 (8D4-8 clone) PE-Cy7 were utilized to identify cytokine secretion. For intracellular cytokine staining, CD95 and CD28 antibodies were stained extracellularly followed by a permeabilization step and CD3, CD4, CD8, IFNγ and IL4 included as intracellular stains. Data was acquired on a BD LSR II and we analyzed stained cell populations using FlowJo (TreeStar).

### MHC inhibition

PBMCs from SIV infected mangabeys were pretreated with antibodies against MHC-I (G46-2.6, BD Bioscience) or MHC-II (Tu39, BD Bioscience) for 2 hrs at 37°C, followed by an incubation with Env peptides (2 µg/ml) in the presence of 2.5 µg of CD28 monoclonal antibody (CD28.2, BioLegend) and 10 µg/ml brefeldin A (Sigma) for 6 hrs. Following stimulation, the cells were then stained with Live-Dead Aqua and surface markers using anti-CD3-APC-Cy7 (clone SP34-2), anti-CD4-PE (clone L200), anti-CD8-PacBlue (clone RPA-T8), anti-CD95-PE-Cy5 (clone DX2) (all from BD Pharmingen) and anti-CD28-ECD (clone CD28.2, Beckman Coulter). After permeabilization, cells were stained intracellular antibodies against anti-Ki67-Alexa700 (clone B56), anti-IL21-Alexa Fluor647 (clone 3A3-N2.1), anti-IFNγ-PE-Cy7 (clone B27) (all from BD Pharmingen); anti-IL17-Alexa Fluor488 (clone eBio64DEC17, eBioscience); anti-IL-2-Brilliant Violet 605 (clone MQ1-17H12, Biolegend). Data was acquired on an LSRII cytometer using the FACS DiVa software and analysis was performed using FlowJo software (TreeStar).

### In vivo BrDU labeling

To assess proliferation and turnover of DN T cells in-vivo, three SIV uninfected and five chronically SIV infected mangabeys were assessed. All animals tested negative for STLV. Infected animals exhibited viral loads greater than 10,000 copies/ml and CD4+ T-cell counts greater than 500 cells/µl. All animals exhibited comparable distributions of T-lymphocyte counts. All animals were treated with intravenously with 60 mg/kg of the thymidine analogue 5-bromo-2′-deoxyuridine BrdU (Sigma-Aldrich) diluted in HBSS (Invitrogen) per weekday and 120 mg/kg orally of BrdU per weekend-day over 14 consecutive days. Cessation of BrDU treatment is the beginning of BrDU washout period.

### Double negative and CD4 T cell isolation

Pure populations of DN and CD4 T cells were isolated by a step-wise magnetic bead isolation (Miltenyi) using beads specific for non-human primate antigens. Briefly, we stained PBMC with CD4-Microbeads to isolate CD4 cells and treated the flow-through with CD8-PE and CD16-PE followed by anti-PE Microbeads for CD8 and CD16 depletion. This CD4/CD8/CD16 depleted flow-through was then positively selected for CD3+ cells with CD3-Biotin and anti-biotin Microbead to obtain, CD3+CD4−CD8− (double negative T cells). Purity was checked after each isolation to guarantee >98% DN T cell pure populations. Cells were rested overnight prior to functional analyses.

### Cell stimulation

We stimulated 1 million purified T cells or PBMC with either anti-CD3 (3 mg/ml) and anti-CD28 (1 mg/ml); or PMA (25 ng/ml) and Ionomycin (1 mg/ml) in 200 ul of complete RPMI at 37°C for 4 hrs (real time PCR and microarray) or 6 hrs (ICS). For Microarray and real time PCR analysis, the cells were collected via centrifugation post-stimulation and immediately lysed in RLT buffer (Qiagen) for RNA extraction. For ICS, the cells were washed and stained appropriately.

### RNA extraction, cDNA synthesis and real time PCR

We isolated RNA (RNeasy Mini Kit – Qiagen) from unstimulated and stimulated CD4 or DN T cells from the same animal and quantified the obtained RNA. cDNA was synthesized using Superscript II First Strand cDNA synthesis kit (Invitrogen) and normalized to 2 ng/ml of starting RNA. Real time PCR was performed utilizing gene-specific primers and probes [74] using the TaqMan system (Applied Biosystem) on the ABI 7300 (Applied Biosystem). We estimated the changes in gene expression between unstimulated and stimulated cells for IFNγ, IL4, IL17, IL10, TGFβ, TNFα and IFNα gene expression (GAPDH - housekeeping gene control) using the ΔΔCt method. Briefly, the Ct value of the specific gene was normalized to the Ct value of GAPDH thus generating a ΔCt value for the gene-of-interest. The ΔCt was calculated by subtracting ΔCt of gene of interest from the unstimulated cells from the ΔCt of gene of interest from the stimulated cells. Fold change for the gene of interest in each cell type was calculated by 2^−ΔΔCt^. Fold change of 1 indicated no upregulation of mRNA upon stimulation.

### Microarray hybridization

Purified DN T cells (magnetic bead isolation) were stimulated for 4 hours with anti-CD3/CD28. Total RNA was isolated from unstimulated and stimulated DN T cells (RNeasy, Qiagen) and genomic DNA was removed on-column. cRNA was synthesized from total RNA and Cy3/Cy5 labeled using the Agilent Low Input Quick Amp Labeling procedure, as per the manufacturer's instructions. Labeled cRNA was hybridized to the Rhesus Macaque (V2) Gene Expression 4×44K Microarray (Agilent) in the appropriate combinations, including dye-flip and self-versus-self controls.

### Microarray analysis

After scanning the arrays, data was background subtracted and the probe signal normalized both within the array and between the arrays, using the *limma* package in Bioconductor [75]. The *limma* averages probe intensity values and fits the values to a linear model, then computes statistics for the data. An adjusted p-value is also computed to correct for multiple testing. A stringent criterion of an adjusted p-value of ≤0.05, in combination with a minimum 4 fold change (log_2_ of 2 or greater) in gene expression between stimulated and unstimulated DN T cells, was used to identify significant differentially regulated genes. Hierarchical cluster analysis was performed using the hierarchical clustering function (Pearson algorithm) in the TM4 Microarray Software Suite [76], [77]. Differentially regulated genes were examined for functional categories using the Database for Annotation, Visualization and Integrated Discovery (DAVID) [78], [79] to elucidate the differential responses between stimulated and unstimulated samples. Network analysis was done using Ingenuity Pathway Analysis (Ingenuity Systems, Inc.).

### Spectratyping

cDNA from purified double negative T cells were amplified with 23 primers specific for the Vβ region of the T cell receptor [42]. Primers were blasted against the Rhesus genome to ensure putative TCR match, we optimized primers 2, 3, 7A, 8, 11, 12B, 14, 17 and 20 by nucleotide substitutions (Primer sequence in Table S1) to ensure complementarity. A single FAM labeled C region (TCR-βC) primer was used as a 3′ primer for each Platinum Taq amplification (Invitrogen). The primers were multiplexed with 4–5 primers per set [42]. Analysis of amplified fragments was done on an ABI fragment analyzer at the Seattle Biomed Sequencing Core and data analyzed on the Peak Scanner. Peak ranges for each Vβ region of the sooty mangabey TCR was identified (Table S1) and expressed Vβ regions along with the extent of junctional diversity was tabulated.

### Data analysis

Graphs were generated using Prism (GraphPad). Data was analyzed identifying Gaussian distribution of differences between sets and using non-gaussian comparison where indicated. A Wilcoxon paired sum test was utilized for paired samples and Mann-Whitney U test for unpaired groups and a p<0.05 considered significant.

## Supporting Information

Figure S1 **Estimation of Vα24 and γδTCR as well as CD4 expression on DN T cells.** A) Flow cytometric analysis of TCR Vα24 (canonical marker of NKT cells) expression assessed on DN T cells from five uninfected (open symbols) and six SIV infected (filled symbols) mangabeys indicates that 0.15% of DN T cells in uninfected mangabeys and 0.2% of DN T cells in SIV infected mangabeys express Vα24. B) Flow cytometric analysis of γδTCR expression on eight SIV infected (filled symbols) and eight uninfected mangabeys (open symbols) indicate that 17% (mean) of DN T cells express the γδTCR. C) PCR amplification of CD4 mRNA in DN and CD4 T cells from SIV+ CD4-healthy, SIV+ CD4-low and uninfected (SIVneg) mangabeys. CD4 mRNA was not detected in purified DN T cells from all the mangabeys groups from both unstimulated (U) cells and cells stimulated (S) with anti-CD3/CD28.
(TIF)

Figure S2 **Longitudinal analysis of DN T cells in rectal mucosa and bronchoalveolar lavage of DN T cells from CD4-low mangabeys.** Flow cytometric estimation of the proportion of DN T cells in A) rectal mucosa and B) bronchoalveolar lavage before and after SIV infection with virally induced dramatic loss of CD4 T cells occurring before day 21.
(TIF)

Figure S3 **Spectratyping of DN T cells.** This figure shows one representative spectratype plot of 3 Vβ regions amplified in a multiplexed PCR reaction from DN T cells. PCR amplified TCRs are visible as peaks quantified on the y-axis by intensity of FAM label. Junctional diversity of each Vβ is seen as multiple peaks amplified from each region, separated by 3 nucleotides (length of PCR product on x-axis). In this DN T cell sample, Vβ 20 was amplified as a clonal peak, Vβ 22 and Vβ 23 demonstrated junctional diversity.
(TIF)

Figure S4 **Quantitative real time PCR analysis of DN and CD4 from uninfected mangabeys upon mitogenic stimulus.** Real time PCR analysis of purified DN and CD4 T cells isolated from 10 uninfected mangabeys was assessed following PMA/Ionomycin (Mitogen) stimulation. DN T cells (filled symbols) upregulate IFNγ, IL4, IL17, TNFα and IL10 at levels similar to CD4 cells (clear symbols) from the same animals. TGFβ and IFNα expression was not altered following TCR stimulation in either DN or CD4 T cells. Log scale fold change is shown on the Y-axis with no change in mRNA expression due to stimulation indicated by a baseline (1 fold).
(TIF)

Figure S5 **Quantitative real time PCR analysis of mitogen stimulated DN and CD4 T cells from SIV infected mangabeys.** Real time PCR analysis of purified double negative and CD4 T cells isolated from SIV infected mangabeys demonstrates upregulation of IFNγ, IL4, IL17, TNFα and IL10 upon stimulation with mitogenic stimulus PMA and Ionomycin. Log scale fold change is shown on the y-axis with no change in mRNA expression due to stimulation indicated by a baseline (1 fold). Cytokine expression of DN T cells from SIV+ CD4-healthy mangabeys (black symbols), SIV+ CD4-low mangabeys (red symbols) and CD4 T cells from SIV+ CD4-healthy mangabeys (open symbols) are depicted. Results demonstrate that DN T cells in SIV infected mangabeys express cytokines at levels similar to CD4 T cells irrespective of SIV-induced CD4 T cell loss.
(TIF)

Table S1 **Primer sequences of 25 Vβ regions amplified.** Primer sequences were based on Rhesus specific TCR. Primer included in each set and final primer concentrations are indicated.
(DOCX)

Table S2 **Junctional diversity of the Vβ amplified during spectratyping.** Table includes number of peaks, peak range and tallest peak are listed for each Vβ amplified
(DOCX)
