# Supplementary figures and images for: Multifunctional Double-negative T Cells in Sooty Mangabeys Mediate T-helper Functions Irrespective of SIV Infection
Source: PLoS Pathog. 2013 Jun 27;9(6):e1003441. doi: 10.1371/journal.ppat.1003441 (PMC3694849; doi:10.1371/journal.ppat.1003441)

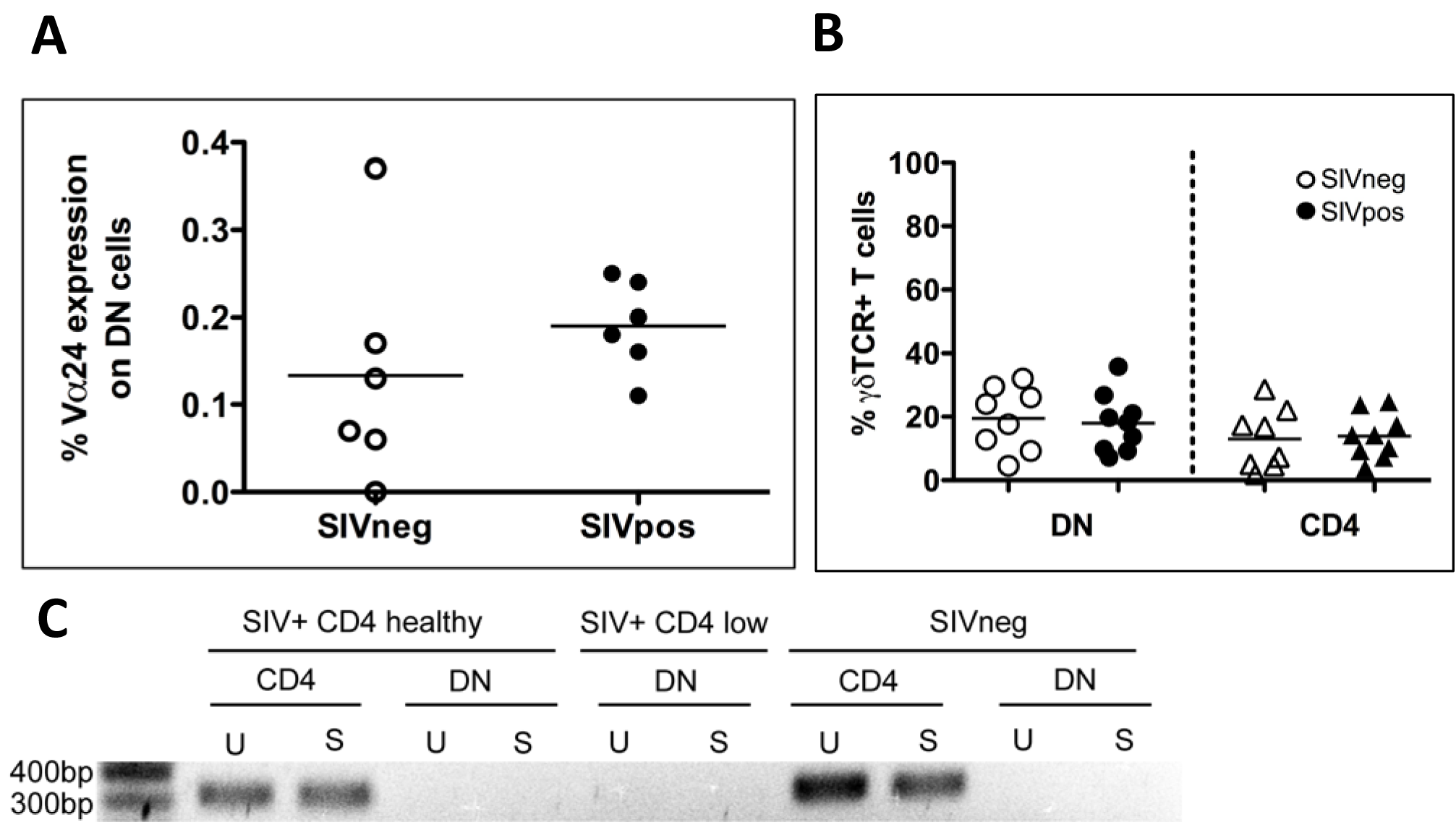

Supplement: Figure S1 — Estimation of Vα24 and γδTCR as well as CD4 expression on DN T cells. A) Flow cytometric analysis of TCR Vα24 (canonical marker of NKT cells) expression assessed on DN T cells from five uninfected (open symbols) and six SIV infected (filled symbols) mangabeys indicates that 0.15% of DN T cells in uninfected mangabeys and 0.2% of DN T cells in SIV infected mangabeys express Vα24. B) Flow cytometric analysis of γδTCR expression on eight SIV infected (filled symbols) and eight uninfected mangabeys (open symbols) indicate that 17% (mean) of DN T cells express the γδTCR. C) PCR amplification of CD4 mRNA in DN and CD4 T cells from SIV+ CD4-healthy, SIV+ CD4-low and uninfected (SIVneg) mangabeys. CD4 mRNA was not detected in purified DN T cells from all the mangabeys groups from both unstimulated (U) cells and cells stimulated (S) with anti-CD3/CD28. (TIF) [file ppat.1003441.s001.tif]

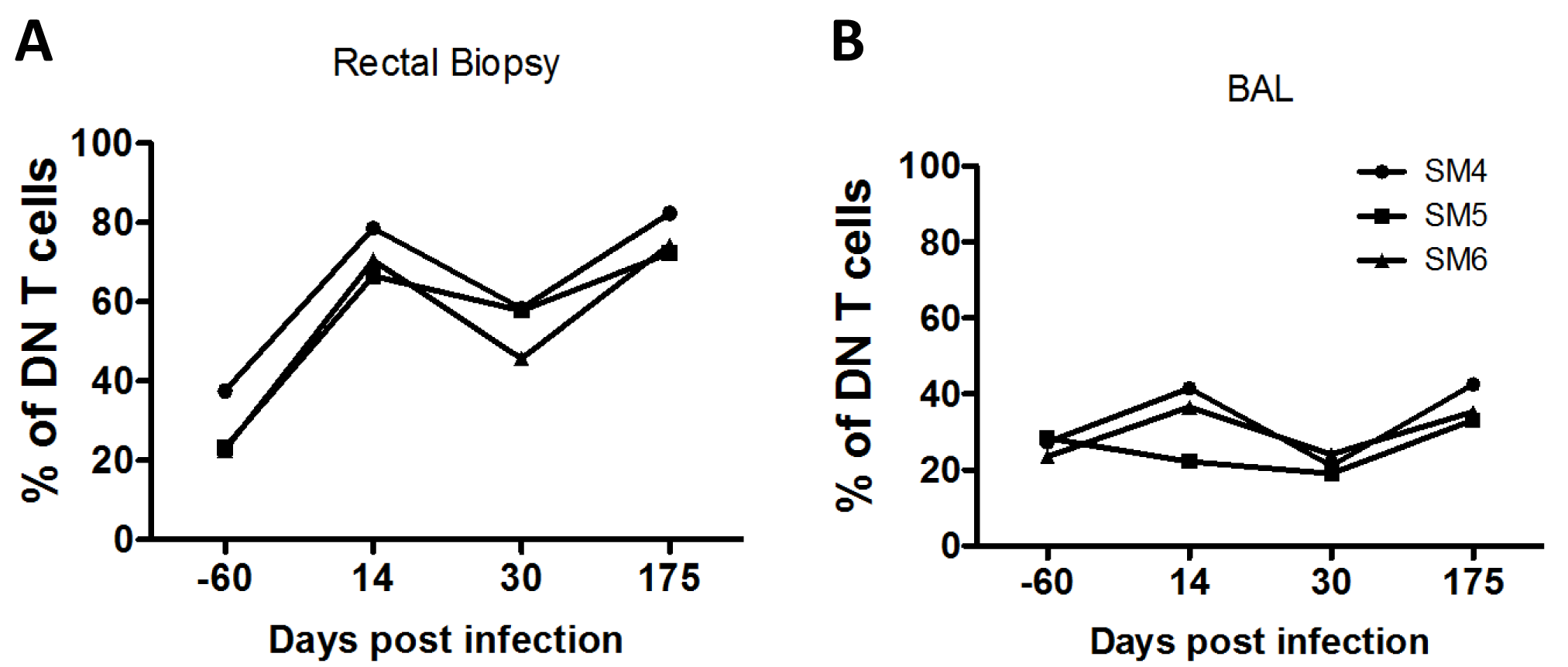

Supplement: Figure S2 — Longitudinal analysis of DN T cells in rectal mucosa and bronchoalveolar lavage of DN T cells from CD4-low mangabeys. Flow cytometric estimation of the proportion of DN T cells in A) rectal mucosa and B) bronchoalveolar lavage before and after SIV infection with virally induced dramatic loss of CD4 T cells occurring before day 21. (TIF) [file ppat.1003441.s002.tif]

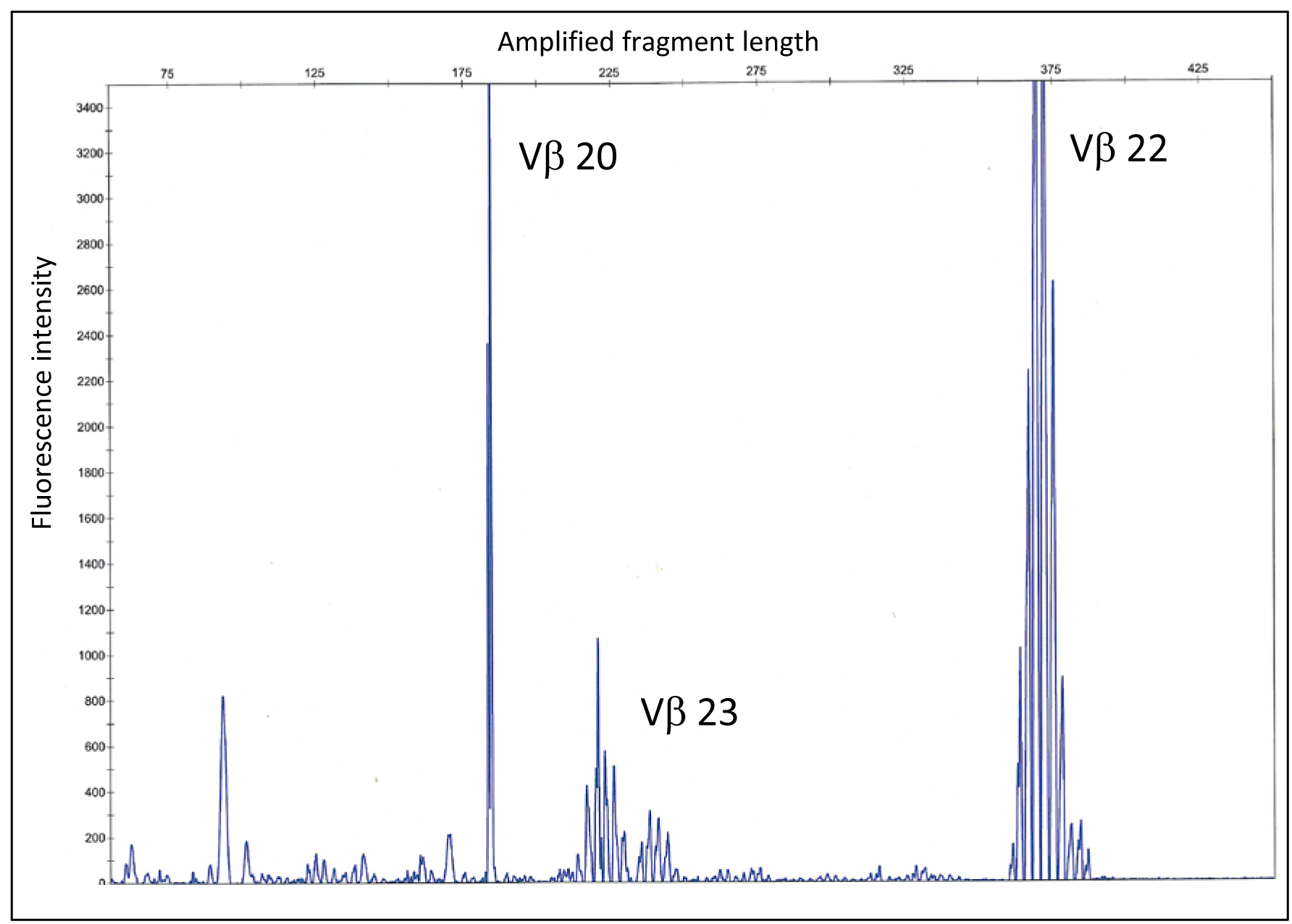

Supplement: Figure S3 — Spectratyping of DN T cells. This figure shows one representative spectratype plot of 3 Vβ regions amplified in a multiplexed PCR reaction from DN T cells. PCR amplified TCRs are visible as peaks quantified on the y-axis by intensity of FAM label. Junctional diversity of each Vβ is seen as multiple peaks amplified from each region, separated by 3 nucleotides (length of PCR product on x-axis). In this DN T cell sample, Vβ 20 was amplified as a clonal peak, Vβ 22 and Vβ 23 demonstrated junctional diversity. (TIF) [file ppat.1003441.s003.tif]

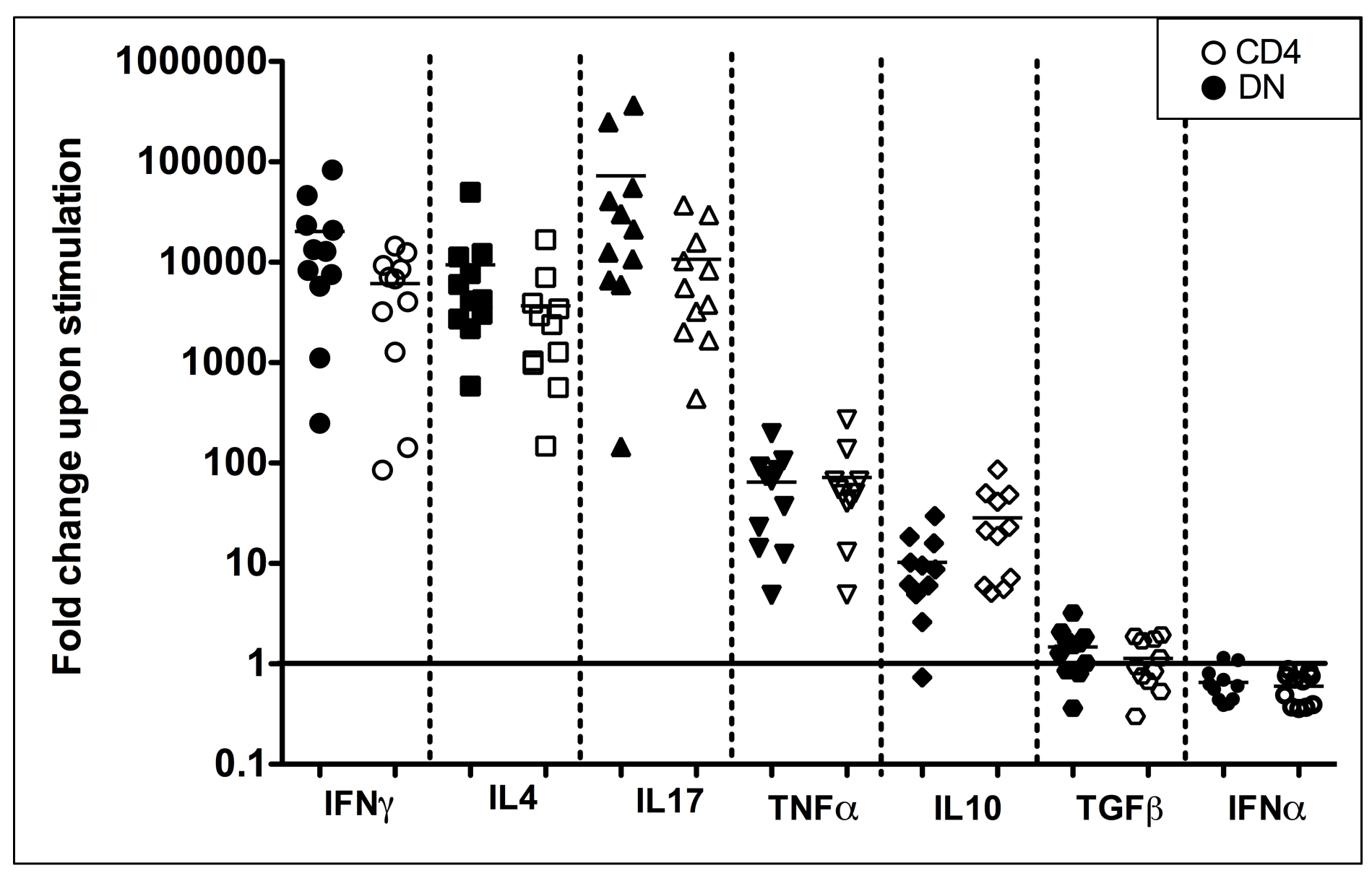

Supplement: Figure S4 — Quantitative real time PCR analysis of DN and CD4 from uninfected mangabeys upon mitogenic stimulus. Real time PCR analysis of purified DN and CD4 T cells isolated from 10 uninfected mangabeys was assessed following PMA/Ionomycin (Mitogen) stimulation. DN T cells (filled symbols) upregulate IFNγ, IL4, IL17, TNFα and IL10 at levels similar to CD4 cells (clear symbols) from the same animals. TGFβ and IFNα expression was not altered following TCR stimulation in either DN or CD4 T cells. Log scale fold change is shown on the Y-axis with no change in mRNA expression due to stimulation indicated by a baseline (1 fold). (TIF) [file ppat.1003441.s004.tif]

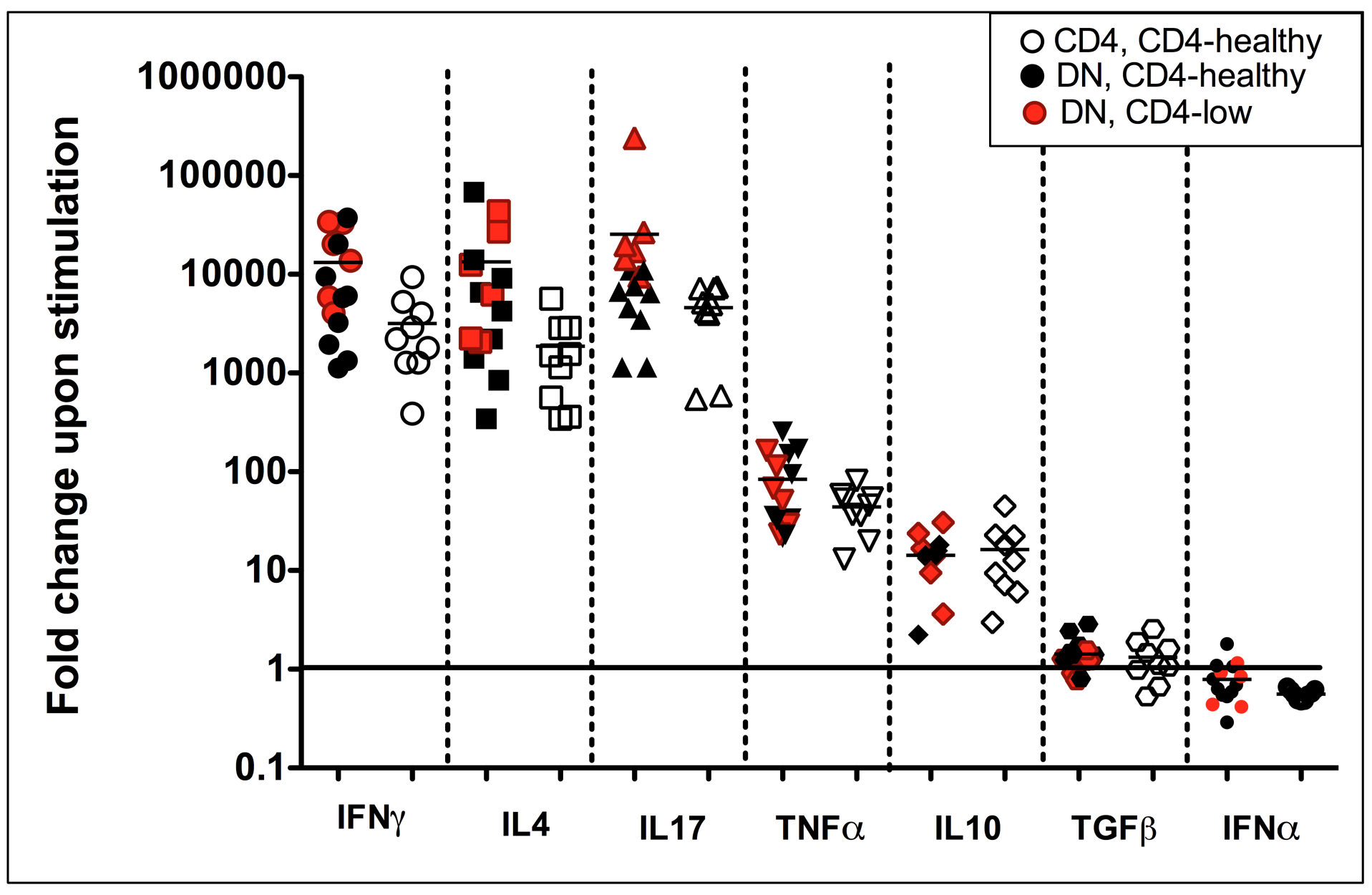

Supplement: Figure S5 — Quantitative real time PCR analysis of mitogen stimulated DN and CD4 T cells from SIV infected mangabeys. Real time PCR analysis of purified double negative and CD4 T cells isolated from SIV infected mangabeys demonstrates upregulation of IFNγ, IL4, IL17, TNFα and IL10 upon stimulation with mitogenic stimulus PMA and Ionomycin. Log scale fold change is shown on the y-axis with no change in mRNA expression due to stimulation indicated by a baseline (1 fold). Cytokine expression of DN T cells from SIV+ CD4-healthy mangabeys (black symbols), SIV+ CD4-low mangabeys (red symbols) and CD4 T cells from SIV+ CD4-healthy mangabeys (open symbols) are depicted. Results demonstrate that DN T cells in SIV infected mangabeys express cytokines at levels similar to CD4 T cells irrespective of SIV-induced CD4 T cell loss. (TIF) [file ppat.1003441.s005.tif]
