# Supplementary material for: Multifunctional Double-negative T Cells in Sooty Mangabeys Mediate T-helper Functions Irrespective of SIV Infection
Source: PLoS Pathog. 2013 Jun 27;9(6):e1003441. doi: 10.1371/journal.ppat.1003441 (PMC3694849; doi:10.1371/journal.ppat.1003441)
Supplement: Table S1 — Primer sequences of 25 Vβ regions amplified. Primer sequences were based on Rhesus specific TCR. Primer included in each set and final primer concentrations are indicated. (DOCX) [file ppat.1003441.s006.docx]

Table S1: Primer sequence for spectratyping analysis.

| Set | TCR-Vβ | Sequence | bp to codon 95 | Final conc |
| --- | --- | --- | --- | --- |
|  | 1 | CTTGCACTCTGAACTAAACC | 67 | 0.06 |
|  | 2 | TGCCGTTCCCTGGACTTTC | 222 | 0.13 |
| 1 | 3 | CAAAGTAACCCAGAGCTCA | 275 | 0.13 |
|  | 4 | ACAGGACAGAGCCTGACA | 171 | 0.13 |
|  | 5 | GACATCAGAAGGAAACTTCCCT | 118 | 0.85 |
|  |  |  |  |  |
|  | 6 | GCTCTCAGGTGTGATCCAA | 225 | 0.37 |
|  | 7A | CACCTTCACCTACACGCCC | 57 | 0.035 |
| 2 | 7B | TBCCTTCACCTACACACCC | 57 | 0.035 |
|  | 8 | ATGCGGGGACTGGAGTTG | 165 | 0.55 |
|  | 9 | AATGAAACAGTTCCAAATCGC | 111 | 0.07 |
|  |  |  |  |  |
|  | 11 | CCAGGAATGGAATTACACC | 165 | 0.14 |
|  | 12A | TGAGATGTCACCAGACTGA | 221 | 0.18 |
| 3 | 12B | TGAGGTGTCACCAGACTTG | 221 | 0.18 |
|  | 13 | ACTCAGACCCCAAAATTCC | 267 | 0.22 |
|  | 13.5 | ATCACCCAGGCACCAACATCT | 270 | 0.1 |
|  | 14 | ATAAGGGAGATATTCCTGAA | 113 | 0.2 |
|  | 15 | ATTCTCCCTGTCCCTAGAG | 62 | 0.23 |
|  |  |  |  |  |
|  | 16 | TCAGTTCCCCAGCCACAG | 268 | 0.13 |
| 4 | 17 | CAGAAAGGAGACATAGCTGAA | 114 | 0.1 |
|  | 18 | GAGGAAGGTCTGAAATTCAT | 165 | 0.13 |
|  | 20 | GGTTCATCCTGAGTTCTAAG | 62 | 0.1 |
|  |  |  |  |  |
|  | 21 | CTCTCAAGATCCAGCCTG | 56 | 0.08 |
|  | 22 | CCAGACTCCCAGCCATCA | 268 | 0.25 |
| 5 | 23 | CAATGCTATCCTATCCCTGA | 216 | 0.13 |
|  | 24 | CAATGAAGCAGACACCCCT | 115 | 0.06 |
|  | 25 | ACAGGTCCTGAAAAACGAGT | 175 | 0.08 |
|  |  |  |  |  |
|  | TCRBC | CTCAAACACAGCGACCTC | - | 1.3 |
