# Supplementary material for: Multifunctional Double-negative T Cells in Sooty Mangabeys Mediate T-helper Functions Irrespective of SIV Infection
Source: PLoS Pathog. 2013 Jun 27;9(6):e1003441. doi: 10.1371/journal.ppat.1003441 (PMC3694849; doi:10.1371/journal.ppat.1003441)
Supplement: Table S2 — Junctional diversity of the Vβ amplified during spectratyping. Table includes number of peaks, peak range and tallest peak are listed for each Vβ amplified (DOCX) [file ppat.1003441.s007.docx]

Table S2: Peak range and junctional diversity for Vβ amplified during spectratyping.

| Vβ | # of Peaks | Peak range (bp) | Tallest peak (bp) |
| --- | --- | --- | --- |
| 2 | 5 | 321-333 | 324 |
| 3 | 7 | 369-385 | 378 |
| 5 | 5 | 216-228 | 222 |
| 6 | 7 | 320-338 | 332 |
| 7A | 5 | 159-168 | 164 |
| 8 | 9 | 267-283 | 273 |
| 9 | 6 | 209-225 | 216 |
| 12B | 7 | 313-322 | 322 |
| 13 | 7 | 363-380 | 372 |
| 15 | 7 | 157-175 | 169 |
| 16 | 6 | 364-379 | 370 |
| 18 | 4 | 271-280 | 277 |
| 20 | 5 | 157-172 | 166 |
| 21 | 6 | 154-170 | 164 |
| 22 | 7 | 362-377 | 368 |
| 23 | 7 | 314-332 | 322 |
| 24 | 7 | 213-228 | 222 |
